# Altered gene expression in *slc4a11*^*−/−*^ mouse cornea highlights SLC4A11 roles

**DOI:** 10.1038/s41598-021-98921-w

**Published:** 2021-10-22

**Authors:** Bernardo V. Alvarez, Marilyse Piché, Carolin Aizouki, Fariha Rahman, Jonathan M. J. Derry, Isabelle Brunette, Joseph R. Casey

**Affiliations:** 1 Department of Biochemistry, Membrane Protein Disease Research Group, University of Alberta, Edmonton, AB T6G 2H7 Canada; 2 Maisonneuve-Rosemont Hospital Research Center, Montreal, Québec Canada; 3 Hansville, USA; 4 Department of Ophthalmology, Université de Montréal, Montreal, Québec Canada; 5 Department of Physiology, University of Alberta, Edmonton, AB T6G 2H7 Canada; 6 Department of Ophthalmology and Visual Sciences, University of Alberta, Edmonton, AB T6G 2H7 Canada

**Keywords:** Biochemistry, Cell biology, Genetics, Molecular biology, Physiology, Molecular medicine

## Abstract

SLC4A11 is a H^+^/NH_3_/water transport protein, of corneal endothelial cells. SLC4A11 mutations cause congenital hereditary endothelial dystrophy and some cases of Fuchs endothelial corneal dystrophy. To probe SLC4A11’s roles, we compared gene expression in RNA from corneas of 17-week-old *slc4a11*^*−/−*^ (n = 3) and *slc4a11*^+*/*+^ mice (n = 3) and subjected to RNA sequencing. mRNA levels for a subset of genes were also assessed by quantitative real-time reverse transcription PCR (qRT RT-PCR). Cornea expressed 13,173 genes, which were rank-ordered for their abundance. In *slc4a11*^*−/−*^ corneas, 100 genes had significantly altered expression. Abundant *slc14a1* expression, encoding the urea transporter UT-A, suggests a significant role in the cornea. The set of genes with altered expression was subjected to Gene Ontology (GO) and Kyoto Encyclopedia of Genes and Genomes (KEGG) pathway analyses, revealing that alterations clustered into extracellular region, cytoskeleton, cell adhesion and plasma membrane functions. Gene expression changes further clustered into classes (with decreasing numbers of genes): cell fate and development, extracellular matrix and cell adhesion, cytoskeleton, ion homeostasis and energy metabolism. Together these gene changes confirm earlier suggestions of a role of SLC4A11 in ion homeostasis, energy metabolism, cell adhesion, and reveal an unrecognized SLC4A11 role in cytoskeletal organization.

## Introduction

Mutations in member 11 of the *S*olute *C*arrier Family *4,* *SLC4A11*, cause congenital hereditary endothelial dystrophy (CHED, OMIM #217700)^1–4^, Harboyan syndrome (HS)^5,6^, and some cases of Fuchs endothelial corneal dystrophy (FECD, OMIM #136800)^7–9^. SLC4A11 is an integral plasma membrane protein highly expressed in basolateral membrane of corneal endothelial cells (CECs)^10^. SLC4A11 has roles in the endothelial cell ion homeostasis and fluid balance by facilitating transmembrane H_2_O movement^11^, Na^+^-independent H^+^ (OH^-^) transport^12^, and NH_3_ transport^13^. More recently, interactions of SLC4A11 with extracellular matrix (ECM) have indicated a role of the protein in cell adhesion^9^.

Until recently CHED was considered to only be caused by *SLC4A11* mutations but a recent publication has added *MPDZ* as a rare CHED gene^14^. FECD is also caused by *SLC4A11* mutations in addition to mutations of *TCF4*, *COL8A2*, *ZEB1*, *AGBL1* and *LOXHD1*, genes^15^. HS is now considered a variant of CHED marked by sensorineural deafness in addition to corneal symptoms. The more than 60 *SLC4A11* missense mutations identified thus far are characterized by two molecular phenotypes: 1) folding defects leading to retention of the protein in the endoplasmic reticulum (ER), with about 50% of the *SLC4A11* missense mutations impairing protein folding and causing ER retention, and 2) defects that impair SLC4A11 protein function^9,16–19^.

CHED and FECD are complex diseases marked by corneal stromal and epithelial edema due to corneal endothelium dysfunction^15^. Cornea is a multilayer structure, composed of an outer epithelium, followed by a connective tissue layer (Bowman’s layer), the stroma (containing interspersed keratocytes), and the basement membrane, Descemet’s membrane (DM) to which the CEC layer adheres. This endothelium separates the corneal stroma from the anterior aqueous humor. CECs actively remove osmotically-accumulated fluid from stroma back into aqueous humor, a process known as the “endothelial fluid pump”^15^. The CEC defects observed in CHED and FECD compromise this pump, leading to stromal edema, corneal hazing and vision loss. A second feature of CHED and FECD is an increased rate of CEC loss, which cannot be compensated by cell proliferation as mature CEC are arrested in the G0-G1 phase of the cell cycle^20^.

Corneal stromal edema and disrupted CEC arrangement^21,22^, pathological features concomitantly found in FECD and CHED patients^15^ and in the *slc4a11*^***−/−***^ mouse model^23^, suggest that loss of SLC4A11 function underlies the pathology associated with *SLC4A11* mutations. Recently, a role of SLC4A11 as a cell adhesion molecule (CAM) contributing to CEC anchorage to the underlying basement membrane, DM, was identified^9^. Defective SLC4A11-DM attachment has been proposed to explain increased CEC loss observed in FECD and CHED patients^9^. DM is a complex meshwork of proteins including collagen I, collagen IV, collagen VIII, fibronectin, vitronectin and laminin^24,25^. CEC cell surface adhesion proteins include heterodimeric integrins alpha-V (αV), beta-3 (β3) and beta-5 (β5)^26^. Moreover, SLC4A11 interacts with main DM components, collagens type VIII alpha chain 1 (COL8A1) and type VIII alpha chain 2 (COL8A2)^9^. SLC4A11 inhibition severely reduced cell adhesion to DM components^9^. Considering these factors, the assumption that cell adhesion defects contribute to FECD and CHED pathology could be inferred.

To understand the cellular pathways linking SLC4A11 protein with symptoms in people affected by FECD and CHED, we investigated the transcriptional profiles of previously characterized *slc4a11*^*−/−*^ mice^11^. Adult *slc4a11*^*−/−*^ mice exhibited macroscopic corneal edema, a progressive increase in corneal thickness, profound disorganization of the corneal endothelium, and CEC swelling, recapitulating key aspects of the progression of human *SLC4A11*-associated diseases^23^. Conversely, a different *slc4a11*^*−/−*^ mouse model showed sensorineural abnormalities and increased corneal thickness with morphologically normal CECs^27^.

In the transcriptome analysis here, we observed gene expression alterations centered on cytoskeletal, membrane-associated, and ECM components in the mouse cornea. This work complements a recent transcriptomic analysis of cultured human CECs subjected to siRNA to reduce their SLC4A11 expression^28^ and analysis of gene expression in human CEC^29,30^. Analysis of gene expression changes compensating for *slc4a11* loss may be useful to identify molecular targets to treat FECD and CHED pathologic conditions.

## Results

### Sequencing results and quality control

Two corneas corresponding to two eyes per animal were collected from three 17 week-old *slc4a11*^+*/*+^ wild type and *slc4a11*^*−/−*^ null mice littermates, respectively. There were three biological replicates in both groups. All the sequencing libraries (six libraries in total) were prepared by the same technician at the same time, and sequenced within a single sequencing run, equally balanced across the flowcell lanes. Trimmed and filtered reads from mouse corneas (following ribosomal transcript removal) were mapped to mouse genome (Ensembl, GRCm38) using hisat2 version 2.0.5^31^. A total of 93–96% reads for all *slc4a11*^+*/*+^ and *slc4a11*^*−/−*^ mouse cornea samples were mapped to the mouse (*Mus musculus*) reference genome (Table 1).

**Table 1:** Sequencing run mapping statistics.

Sample ID	Total reads	Mapped reads	% of mapped reads
*slc4a11*^+*/*+^ 1	24,689,292	23,289,622	94
*slc4a11*^+*/*+^ 2	19,193,868	18,485,986	96
*slc4a11*^+*/*+^ 3	3,457,512	3,272,941	95
*slc4a11*^*−/−*^ 1	22,441,611	20,975,724	93
*slc4a11 *^*−/−*^ 2	20,761,849	19,899,296	96
*slc4a11*^*−/−*^ 3	9,316,547	8,779,019	94

Percentage of trimmed and filtered reads successfully mapped to the genome, from total reads sequenced for each sample.

### Cluster and principal component analysis of altered gene expression

Genes with low expression were removed from analysis with the following criteria. Genes with CPM equal or over 1 in at least 2 samples were retained in the analysis. The genes that were removed had an average transcript per million (TPM) of 0.31. The filtering resulted in the removal of 46,078 of annotated features with low or absent expression while 13,565 were retained for the further analysis. Distribution of normalized and variance stabilized gene expression values across individual sequencing libraries revealed similar characteristics for RNA isolated from each of the six mice (Suppl. Figure 1A). Relationships between samples were explored by non-supervised hierarchical cluster analysis visualized as heatmaps (Suppl. Figure 2), and principal component analysis (PCA) visualized as principal component plots (Suppl. Figure 1B). Clustering and PCA were performed on the 1500 genes with the highest median absolute deviation (MAD) in expression in *slc4a11*^*−/−*^ versus *slc4a11*^+*/*+^ mouse corneas. In the case of hierarchical clustering, the distance measure was Euclidean and clustering algorithm (ward D). Biological replicates clustered closely in hierarchical clustering and PCA (Suppl. Figure 2 and Suppl. Figure 1B), which reflected the similarity of their gene expression profiles.

### Differential expression analysis

**Figure 1:**
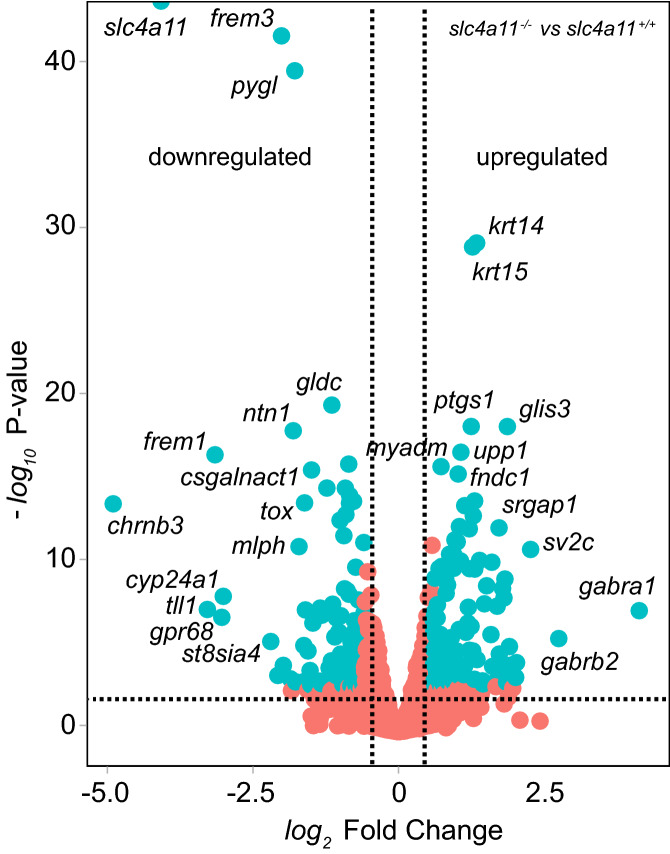
Gene expression profiles for s*lc4a11*^+*/*+^ and *slc4a11*^*−/−*^ corneal RNA samples. Genes significantly down and up regulated in *slc4a11*^*−/−*^ cornea samples are indicated by blue dots. Genes with no significant differential expression are represented by red dots. Dashed lines indicate the boundaries for genes with significantly altered expression. The x-axis represents fold change of gene expression, and the y-axis represents the magnitude of significance of the gene expression changes. Identified gene examples: *slc4a11,* solute carrier family member 11; f*rem3*, fras 1 related extracellular matrix protein 3; *ntr1*, netrin 1; *pygl*, prostaglandin endoperoxidase synthase 1; *frem1*, fras 1 -related extracellular matrix 1; *csgalnact1*, chondroitin sulfate N-acetylgalactosaminyltransferase-1; *tox*, thymocyte selection-associated high mobility group box; *mlph*, melanophilin; *tll1*, tolloid-like protein 1; *gpr68,* G-protein-coupled receptor 68; *st8sia4*, polysialyltransferase; *upp1,* uridine phosphorylase 1; *ptgs1,* liver glycogen phosphorylase 1; *krt 14*, keratin; *krt* *15*, keratin; *glis 3*, glis family zinc finger 3; *myadm*, myeloid associated differentiation marker; *fndc1*, fibronectin type III domain containing 1; *srgap1*, slit-robo GTPase-activating protein; *sv2c*, synaptic vesicle glycoprotein 2; *gabra1*, gamma-aminobutyric acid receptor A subunit; *gabrb2*, gaba receptor B subunit.

Normalization of transcript reads (number of times a given gene was identified amongst the sequenced transcripts) was conducted using DESeq () function with default parameters. Cornea genes with adjusted P value less than 0.05 and the fold change over 1.5 were considered differentially expressed (Fig. 1). The gene expression differences are well visualized on a MA plot (Bland–Altman plot), which transforms the RNAseq data onto M (*log* ratio) and A (mean average) scales (Fig. 1). The results were annotated with gene symbols, entrez identifications numbers and gene descriptions using biomaRt 2.40.3 Bioconductor package. In each case, expression changes are reported relative to the *slc4a11*^*−/−*^ mice (samples 1–3) (Suppl. Table 1). Amongst the 1500 genes with the highest median absolute deviation, 100 were significantly differentially expressed in *slc4a11*^*−/−*^ mouse corneas compared to *slc4a11*^+*/*+^ (absolute value of log_2_ ratio ≥ 1, adjusted p-value < 0.05). The list of genes with significantly altered expression is in Suppl. Table 1. Comparing *slc4a11*^*−/−*^ and *slc4a11*^+*/*+^ gene expression data, 57 and 43 genes were significantly over and under-expressed, respectively (Suppl. Table 1). To identify transcripts expressed in CEC, transcripts previously identified in human CEC^29,30^ were compared to the list of differentially expressed genes. Amongst the 100 genes with significantly altered expression, 68 are expressed in human CEC (Suppl. Figure 1). The 32 genes not expressed in CEC are thus likely expressed in the cornea’s other cell types, predominantly epithelial cells and stromal keratocytes, together suggesting that *slc4a11* loss has effects beyond CEC. We are unable to identify which of the remaining 68 significant gene expression changes has occurred in CEC versus other cell types. Nonetheless, compensatory gene expression changes do point toward processes affected by *slc4a11* loss in the cornea. Alternatively, the lack of expression in human CEC may reflect isoform differences between species. Clustering of differentially expressed genes using the Euclidean distance method-clustering algorithm (ward D) revealed clusters of differentially expressed genes between the *slc4a11*^+*/*+^ and *slc4a11*^*−/−*^ mouse groups (Suppl. Figure 3).

### Gene ontology (GO) and pathway analyses of genes with significantly altered expression in *slc4a11*^*−/−*^ mouse cornea

**Figure 2:**
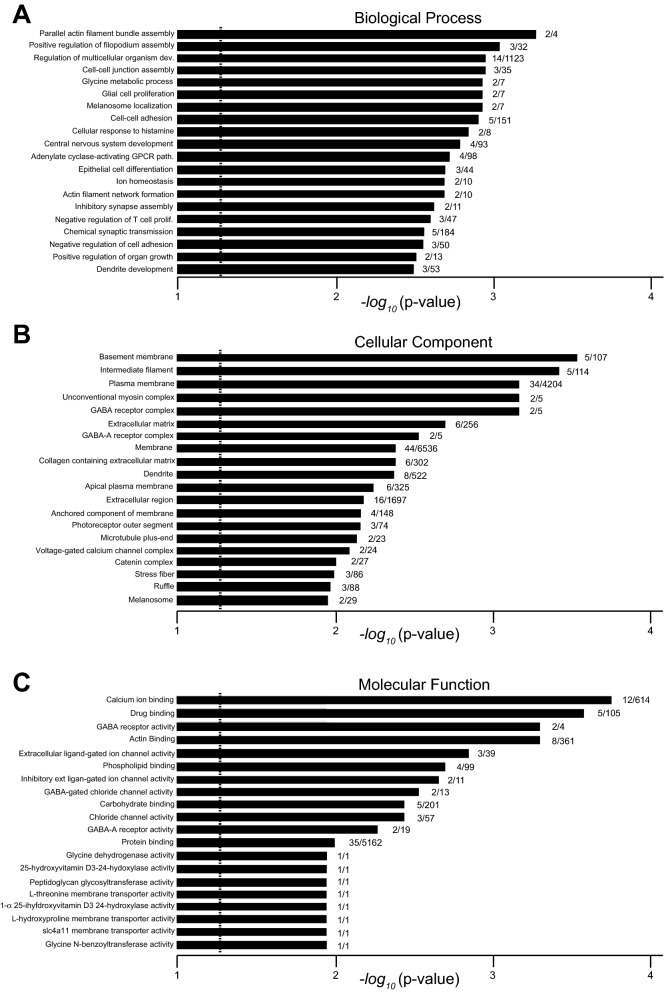
Gene ontology (GO) analysis of genes differentially expressed in *slc4a11*^*−/−*^ mouse cornea. The X-axis represents the rank of GO terms based on statistical significance of the alterations of gene expression for genes in a GO category. Numbers beside bars are number of altered genes identified in the pathway/ number of genes in that GO pathway total. Top 20 affected GO category are indicated for: (**A**) biological process, (**B**) cellular component, and (**C**) molecular function.

Two unbiased approaches were used to identify patterns in the genes with significantly altered expression in *slc4a11*^*−/−*^ mouse corneas: GO and pathway analyses. GO analysis of the 100 significantly altered genes revealed Extracellular region, Cytoskeleton, Cell Adhesion and Plasma membrane as the highest gene clusters (Table 2). Amongst biological processes, actin filament assembly, filipodium assembly, developmental regulation and cell–cell junction assembly were the most abundant terms (Fig. 2A). Similarly, between cellular component terms, most abundant were basement, intermediate filament, plasma membrane, and ECM components (Fig. 2B). Clustering of gene alterations by molecular function revealed that membrane processes, including calcium ion binding, drug binding and actin binding, were also significantly enriched (Fig. 2C).

**Table 2:** Gene ontology (GO) enrichment analysis.

Gene	Protein encoded	Fold change	Gene	Protein encoded	Fold change
**Extracellular region (GO0005576)**					
*Mmrn1*	Multimerin 1	4.0	*Matn2*	Matrilin 2	2.1
*Sostdc1*	Sclerostin domain containing 1	3.8	*Tll1*	Tolloid-like	0.10
*Spink5*	Serine peptidase inhibitor, Kazal 5	3.5	*Frem1*	Fras1 related ECM 1	0.11
*Dkk1*	Dickkopf WNT pathway inhibitor 1	2.9	*Frem3*	Fras1 related ECM 3	0.25
*Col14a1*	Collagen, type XIV, alpha 1	2.7	*Ntn1*	Netrin 1	0.29
*Loxl2*	Lysyl oxidase-like 2	2.3	*Pla2g2f.*	Phospholipase A2, group IIF	0.29
*B3gat1*	B-1,3-glucuronyltransferase 1	2.2	*Tnfrsf11b*	Tnf receptor 11b	0.33
*Lgals7*	Lectin, galactose binding, soluble 7	2.2	*C4b*	Complement component 4B	0.35
**Cell adhesion (GO0007155)**					
*Col14a1*	Collagen, type XIV, alpha 1	2.73	*Frem3*	Fras1 related ECM 3	0.25
*Cdh3*	Cadherin 3	2.33	*Fat4*	Fat atypical cadherin 4	0.44
*Cdh13*	Cadherin 13	2.32	*Ripor2*	RHO family interacting cell polarization regulator 2	0.46
*Frem1*	Fras1 related ECM 1	0.11			
**Cytoskeleton (GO0005856)**					
*Tubb4a*	Tubulin, beta 4A class IVA	3.32	*Stk39*	Serine/threonine kinase 39	2.37
*Fscn1*	Fascin actin-bundling protein 1	3.01	*Ppp1r18*	Protein phosphatase 1, regulatory 18	2.02
*Fhod3*	Formin homology dom. containing 3	2.63	*Espn*	Espin	0.25
*Map1b*	Microtubule-associated protein 1B	2.44	*Ripor2*	See above	0.46
**Plasma membrane (GO0005886)**					
*Gabra1*	GABA A receptor, alpha 1	17.58	*Smo*	Smoothened receptor	2.01
*Gabrb2*	GABA A receptor, beta 2	6.74	*Chrnb3*	Cholinergic R., nicotinic, b- 3	0.03
*Vtcn1*	V-set containing T cell activation inhibitor 1	3.24	*Slc4a11*	Solute carrier family 4 member 11	0.06
*Fscn1*	Fascin actin-bundling protein 1	3.01	*Gpr68*	GPCR 68	0.12
*Dlk2*	D like non-canonical Notch ligand 2	2.78	*Cyp24a1*	Cytochrome P450, family 24, 1	0.12
*Dkk1*	Dickkopf WNT signaling pathway inhibitor 1	2.86	*Hif3a*	Hypoxia inducible factor 3, alpha	0.24
*Myadm*	Myeloid-associated differentiation marker	2.48	*Pla2g2f*	Phospholipase A2, group IIF	0.29
*Map1b*	Microtubule-associated protein 1B	2.44	*Cntfr*	Ciliary neurotrophic factor R	0.39
*Pth1r*	Parathyroid hormone 1 receptor	2.42	*Clec2g*	C-type lectin domain 2, g	0.39
*#Cdh3*	Cadherin 3	2.33	*Vnn1*	vanin 1	0.43
*Cdh13*	Cadherin 13	2.32	*Cacnb2*	Ca^++^ channel, voltage-dependent, b 2	0.44
*Adcy3*	Adenylate cyclase 3	2.17	*Fat4*	Fat atypical cadherin 4	0.44
*Gpr161*	gpcr 161	2.04	*Gldc*	Glycine decarboxylase	0.45
*Fndc1*	Fibronectin III domain containing 1	2.04	*Ripor2*	See above	0.46
*Adgrg2*	Adhesion GPCR G2	2.00			

Genes with significantly altered expression were subjected to gene ontology analysis. GO categories name and code are in the table in bold.

*GABA*  gamma amino butyric acid, *GPCR* G protein coupled receptor, *ECM*  extracellular matrix, *TNF* tumor necrosis factor, *R*  receptor, *Dom.*  domain.

**Figure 3:**
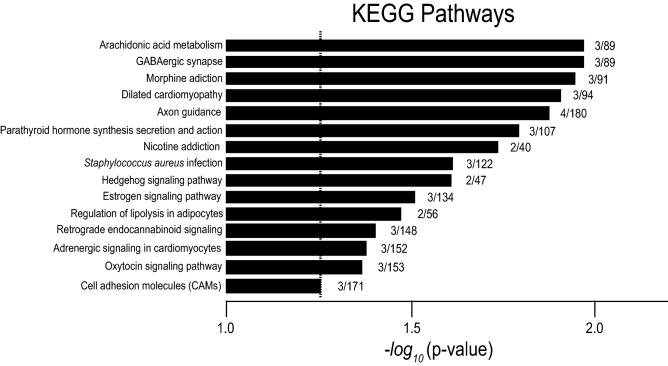
Kyoto Encyclopedia of Genes and Genomes (KEGG)^4^ pathways significantly enriched amongst differentially expressed genes. The X-axis represents the rank of KEGG terms based on statistical significance of the alterations of gene expression for genes in each category. Numbers beside bars are number of altered genes identified in the pathway/number of genes in that KEGG pathway total. Top 20 affected GO pathways are indicated.

Signaling pathways were analyzed by the Kyoto Encyclopedia of Genes and Genomes (KEGG)^4^. KEGG analysis revealed significantly affected pathways in *slc4a11*^*−/−*^ mice (Fig. 3, including the arachidonic acid metabolism pathway (mmu00590), the GABAergic synapse pathway (mmu04727), the estrogen signaling pathway (mmu04915), and hedgehog signaling (mmu04340). The latter pathway importantly is associated with cornea development and cell proliferation-associated healing processes^32^. Other pathways altered in *slc4a11*^−/−^ mouse cornea that may be compensatory mechanisms include the cell adhesion molecules (CAMs, mmu04514) and the gap junction (mmu04540), likely to be abnormally activated during stress processes occurring in the cornea.

### Subjective analysis of expression changes

In addition to the unbiased analysis of genes with altered expression in *slc4a11*^*−/−*^ mouse cornea (GO and KEGG analysis above), we also considered the significance of gene alterations in the context of corneal dystrophy biology. The function of differentially expressed genes was determined by literature review (Suppl. Table 1). Six categories of genes were identified (ordered from most to fewest genes in each class): Cell fate/development, Extracellular matrix/ cell adhesion, Cytoskeleton, Ion homeostasis and fluid handling, Energy metabolism and Vesicular trafficking (Table 3). Fourteen genes remained uncategorized as they did not fall clearly into any of the classes. Interestingly, there may be additional subclasses amongst these 14, with some evidence for a group of prostaglandin metabolizing genes (cyclooxygenase 1 (ptgs1), prostaglandin E synthase (*ptges*), phospholipase 2g2f (*pla2g2f*)) and nucleoside metabolism and binding (ecto-5’-nuclotidase (*nt5e*), adenylate cyclase (*adcy3*), uridine phosphorylase 1 (*upp1*), guanylate binding protein (*gbp8*)). This analysis was compared to an objective Gene Ontology (GO) classification. GO recognized four categories including extracellular region, cytoskeletal, cell adhesion, and plasma membrane. Three of the GO categories matched with sections identified through the subjective review of genes including cytoskeleton, ECM, and cell adhesion.

**Table 3:**
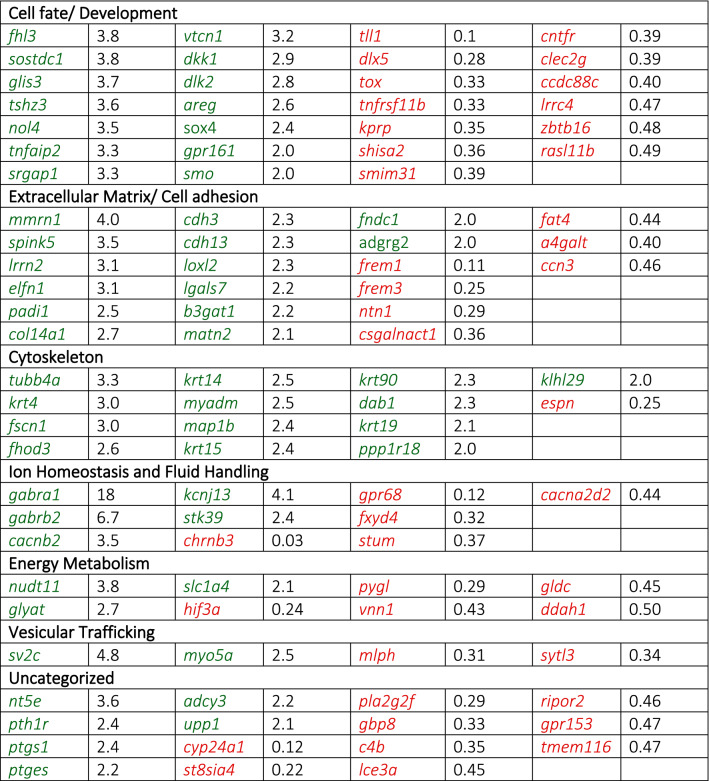
Organization of genes with altered expression into functional categories.

Genes with increased expression are written in green text and red indicates decreased expression. Genes in the table are ordered from most to least altered in both the increased and decreased categories. To the right of each gene name are fold changes indicating change of expression in *slc4a11*^*−/−*^ mice relative to *slc4a11*^+*/*+^*.* Additional information on the role of each gene and the significance of each alteration can be seen in Suppl Table 1. Functional categories are indicated in bold in the table. Categories are ordered by number genes in each category.

### Corneal gene expression

**Figure 4:**
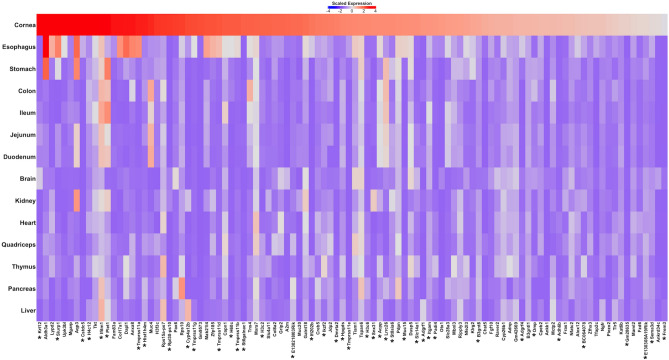
Mouse corneal gene expression. Gene expression data from RNAseq were processed to normalize and identify the 102 most abundant transcripts in mouse cornea. Expression of these genes was compared to expression levels of these genes in a panel of 13 mouse tissues (from tissue expression database, https://www.omicsdi.org/dataset/arrayexpress-repository/E-MTAB-6081). Heat map illustrates relative expression level (see inset scale) in cornea and across indicated tissues. * indicates genes not identified as expressed in human CEC (on the basis of genes expressed in isolated human CEC^29,30^) and thus likely expressed in corneal epithelial cells. One of these studies measured transcript abundance in 15 human CEC samples^30^. In this case, we considered genes as expressed in CEC if they were found in 10/15 samples.

In addition to assessing gene expression changes occurring with loss of *slc4a11*, RNAseq data provide information on the genes expressed and the relative abundance of their respective mRNAs. Data averaged from three *slc4a11*^+*/*+^ mice revealed expression of 13,565 genes, of 25,059 protein coding genes in the mouse genome^33^. To place the gene expression data in physiological context, corneal gene expression was compared to expression data for a set of mouse tissues. Principal component analysis clustered the *slc4a11*^*−/−*^ and *slc4a11*^+*/*+^ expression patterns most closely with epithelial tissues, including stomach, colon, ileum, duodenum, and jejunum and most different from brain, muscle, and liver (Suppl. Figure 4), which one would expect from corneal physiology. To begin to understand this large dataset, we plotted the expression level of the 101 most abundant transcripts in mouse cornea, in comparison to 13 other tissues (Fig. 4). Amongst these 101 genes, 41 were not found to be expressed in human CEC^29,30^ (Fig. 4).

### Assessment of gene expression by quantitative real-time RT-PCR

Validity of RNAseq results was assessed on a subset of genes, monitoring expression levels by quantitative Real Time RT-PCR (qRT RT-PCR). Top overexpressed and downregulated genes separated in categories were chosen to test using qRT RT-PCR. Additional genes were selected for analysis: *gapdh* was as a control gene for normalization, and FECD genes, (*tcf4*, *col8a2*, and *lamc1*), whose expression did change in *slc4a11*^*−/−*^ corneas.

Reliability of qRT RT-PCR detection was assessed by electrophoresis of PCR products on agarose gels (Suppl. Figure 5), which revealed single PCR products of the expected size, indicating specific amplification. Since two bands appeared on the gel for *col14a1* (Suppl. Figure 5A), a separate set of samples was analyzed on agarose gels with a clear single band for *col14a1* expression in the *slc4a11*^*−/−*^ mouse cornea sample (Suppl. Figure 5B). However, no band was detected in *slc4a11*^+*/*+^ samples, which expresses very low levels of *col14a1* as determined by RNAseq analysis (Suppl. Figure 5B). We confirmed *col14a1* amplification using isolated mouse heart mRNA qRT RT-PCR (not shown). Melting curves were generated for each gene amplified, which revealed single, specific products. Similarly, melting curves for *tubb4a* and *espn* genes with low expression levels showed single peaks, therefore revealing specific products (Suppl. Figure 5C).

**Figure 5:**
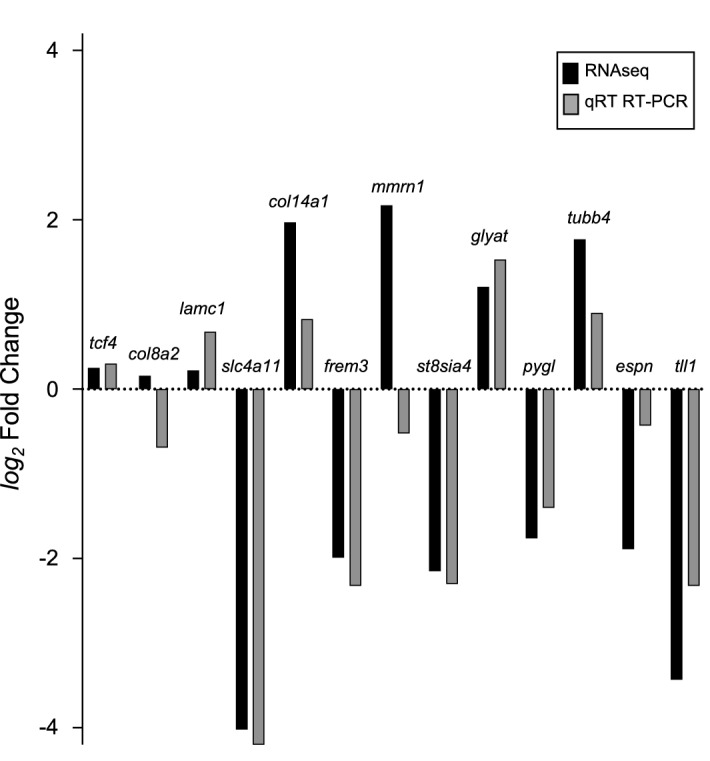
Comparison of RNAseq and qRT RT-PCR data. Mean values of RNAseq and qRT RT-PCR corresponding to different genes analyzed on *slc4a11*^+*/*+^ and *slc4a11*^*−/−*^ mice cornea were converted to log_2_ fold change and used to verify the two methods.

qRT RT-PCR revealed that all genes had comparable results with RNA-Seq data (Fig. 5). Genes with increased expression in *slc4a11*^*−/−*^ cornea by RNA-Seq (glyat, *col14a1, tubb4*, *tll1*), also gave increased expression relative to *gapdh* following qRT RT-PCR. Likewise, genes with decreased expression in *slc4a11*^*−/−*^ (*frem3*, *st8sia4*, *pygl;* Suppl. Table 1) had decreased expression by qRT RT-PCR (Fig. 5). Espin (*espn*) followed the same expression change pattern by RNAseq and qRT RT-PCR analysis, without reaching statistically significant differences by qRT RT-PCR (Fig. 5). Interestingly, *lamc1* was overexpressed in *slc4a11*^*−/−*^ cornea as measured by qRT RT-PCR (Fig. 5), while *lamc1* expression was slightly increased (P < 0.058) in *slc4a11*^*−/−*^ null mice compared to *slc4a11*^+*/*+^ mouse cornea, by RNAseq analysis. *mmrn1* expression changes went in slightly different directions by RNAseq and qRT RT-PCR (Fig. 5). Similarly, two genes whose expression was not significantly altered in RNA-Seq results, *tcf4* and *col8a2*, were also unaltered when assessed with qRT-PCR (Fig. 5). Taken together, the RT-PCR data validated the changes seen by RNAseq.

## Discussion

*SLC4A11* mutations are responsible for CHED^1–3^, HS^5,6^, and some cases of late-onset FECD^7–9,15^. To better understand the role of SLC4A11 protein in corneal dystrophies, we took advantage of a previously characterized *slc4a11*^*−/−*^ mice to perform a transcriptome analysis of adult mouse cornea. These data provide additional support for roles of SLC4A11 in ion homeostasis, energy metabolism and cell adhesion. A role of SLC4A11 in cytoskeletal organization is also uncovered by the data here. Expression analysis also revealed three genes with possible important unexplored roles in corneal epithelial cells (*slc14a1*) and in hearing deficits (*espn and ripor2)* associated with *SLC4A11* mutations.

Among the 100 significantly altered genes in *slc4a11*^*−/−*^ cornea, the largest cluster (27 genes; Table 3, Suppl. Table 1) have roles in cell fate determination and development. Broadly these have roles as transcription regulators (*fhl3*, *glis3*, *tshz3*, *sox4, dlx5*, *tox*, *zbtb16*), developmental signaling pathway components (*sostdc1*, *srgap1*, *vtcn1*, *dkk1*, *dlk2*, *areg*, *gpr161*, *smo*, *tll1*, *tnfrsf11b*, *smim31*, *shisa2*, *cntfr, rasl11b*), and cell fate specificity factors (*nol4*, *tnfaip2*, *kprp*, *ccdc88c*, *lrrc4, clec2g*). These changes reveal that *slc4a11* loss profoundly affects cell organization and phenotype, but how so? It may be that SLC4A11 has a key role as part of the endothelial fluid pump, which when compromised leaves cells in a state requiring major changes. Failed glutamine metabolism has been suggested to arise with slc4a11 loss, which could lead to energy deficits, giving rise to phenotypic changes. Finally, SLC4A11 was recently found to contribute to CEC adhesion to ECM (Descemet’s membrane)^9^. Cell adhesion is critical to cells being able to understand their cell type and fate. Thus, disruptions of cell adhesion could potentially underlie the alterations of cell fate and developmental genes seen here.

The second most abundant class of gene alterations observed was ECM and cell adhesion. Of the 100 genes with significant expression changes 21 were in this class. Among genes in this class whose expression increased were genes involved in bridging cell surface adhesion proteins to ECM (*mmrn1, elfn1, lgals7, b3gat1*), cell surface adhesion proteins (*lrrn2, cdh3, cdh13, adgrg2*), ECM post translational modifying enzymes (*padi1*, *loxl2*), ECM proteins (*col14a1, matn2, fndc1*), a secreted serine protease inhibitor (*spink5*). Conversely, in this gene class with reduced expression were genes involved in bridging cell surface adhesion proteins to ECM (*frem1, frem3, ntn1, fat4, a4gal*), ECM post translational modifying enzyme (*csgalnact1*), and regulator of ECM gene expression (*ccn3*). Increased expression of cadherins 3 and 13, in addition to proteins bridging cell surface to ECM are consistent with a loss of cell adhesion capacity upon deletion of *slc4a11* and is in line with the recently identified role of SLC4A11 as a cell surface adhesion protein^9^. Intriguing observations that SLC4A11 induces integrin clustering to induce focal adhesion formation and that SLC4A11 co-localizes with fibronectin-binding integrins additionally support a role of SLC4A11 in coupling cells to ECM^34^.

Among the 100 genes with significantly altered expression in *slc4a11*^*−/−*^ corneas (Suppl. Table 1), 14 encoded cytoskeletal proteins. Among these were five keratin genes, which may indicate a role in corneal epithelium not endothelium. Indeed, amongst these five, only *KRT15* and *KRT19* are expressed in human CEC^30^ (Suppl. Table 1). Increased expression of nine cytoskeletal protein genes, however, suggests that cells are attempting to compensate for loss of cytoskeletal integrity that arises upon loss of *slc4a11*. Among these, were increased expression of genes encoding beta tubulin, a component of microtubules, fascin, an actin bundling protein important in focal adhesions, actin organizing protein, *fhod3*, *myadm*, an integral membrane protein that organizes interaction between cytoskeleton and the plasma membrane, *map1b, a* microtubule assembly protein, *dab1*, an intracellular signaling protein important in nectin/cadherin/fibronectin adhesion, and *ppp1r18*, which negatively regulates actin ring formation. The only cytoskeletal protein gene with reduced expression was *espn*, which encodes a protein that regulates actin filament function. The picture that emerges from these gene changes is a cell adapting to altered cytoskeletal dynamics. This may reflect the cell swelling that could develop secondary to fluid handling defects.

Alternatively, SLC4A11 may have a previously undescribed role in the cytoskeleton. Communication between ECM and cytoskeleton is critical and requires integral membrane proteins, like SLC4A11. Cytoskeletal protein binding has not been reported for SLC4A11, but the protein has a 41 kDa cytoplasmic domain with poorly understood function^35^. A role in cytoskeletal binding is well established for SLC4A1 (Band 3/AE1), giving strong precedence for this possibility in the SLC4 family. Taken together, the observed changes of cytoskeletal changes suggest a role of SLC4A11 in cytoskeletal protein binding, perhaps linking ECM to cytoskeleton.

The most important role of the CEC layer is “fluid pumping” or accumulated fluid from the corneal stroma back to aqueous humor^15^. Eight CEC genes involved in ion homeostasis and fluid handling were identified with altered expression in *slc4a11*^*−/−*^ mouse corneas. SLC4A11 functions as a plasma membrane H^+^ channel, which has been proposed to neutralize the alkalinizing effect of HCO_3_^-^ accumulation via the electrogenic Na^+^/HCO_3_^-^ co-transporter, NBCe1, as part of the endothelial fluid pump^3^. In *slc4a11*^*−/−*^ mice, the acidifying activity of SLC4A11 is lost. CEC may compensate by increased activity of the AE2 Cl^-^/HCO_3_^-^ exchanger, present in CEC, which acidifies cells by efflux of HCO_3_^-^ in exchange for influx of Cl^-^. Although AE2 *(slc4a2*) mRNA was unaltered in *slc4a11*^*−/−*^ corneas, its expression was detected. AE2 has a high transport rate^36^, enabling compensation for *slc4a11* loss without requiring increased expression.

In reviewing alterations in *slc4a11*^*−/−*^ mouse cornea, the absence of changes in pH-regulatory transporter gene expression is striking. Interestingly, expression of *gpr68*, an acid sensing G protein coupled receptor, decreased, which might be expected in a cell undergoing acid–base regulation abnormalities. If SLC4A11 is required to prevent corneal dystrophy through NH_3_/H^+^/OH^-^ transport, then compensation by altered expression of ion transporters would be expected. SLC4A11 transports H^+^/OH^-^ and NH_3_, yet loss of *slc4a11* did not lead to increased expression of other acid–base transporters (e.g., *mct1*, *ae2*, *nbce1*). This suggests that SLC4A11’s critical indispensable role is not plasma membrane solute transport.

Possible deficits in the CEC “fluid pump”, however, are indicated by increased *stk39* expression. STE20 (sterile 20-like)-related proline-alanine-rich kinase (SPAK) is encoded by *stk39*. SPAK kinases are important in osmotic stress signalling so that *stk39* upregulation might reflect a cell attempting to compensate for the osmotic stress that might be expected with failing fluid pumping.

Outside of CEC, the large changes of GABA receptor (*gabra1* and *gabrb2*) expression may arise in Schwaan cells associated with corneal nerves, especially since these genes are not expressed in human CEC^30^. Although GABA receptors are expressed in glia^37^, the expression of SLC4A11 in neurons or glia and physiological reasons for SLC4A11 function in these cells have not, however, been studied.

SLC4A11 defects are recognized to manifest with compromised energy metabolism. In particular, strong evidence supports a role of SLC4A11 in facilitating CEC glutaminolysis in energy production^28,38,39^ and suggested a role of SLC4A11 in uncoupling mitochondria to prevent oxidative damage^40^. In the present study, we identified seven expression alterations related to CEC energy metabolism (Table 3, Suppl. Table 1). Increased expression was observed for: 1. *Nudt11*, an enzyme that produces the glycolytic activator, ribose 1.5 bisphosphate, 2. *Slc1a4*, a sodium-dependent neutral amino acid transporter responsible for cellular glutamine uptake. In *slc4a11*^*−/−*^ mice, expression decreased for: 3. Hypoxia inducible factor 3 alpha subunit (*HIF3a*), critical for response to hypoxia and transition to anaerobic metabolism, 4. Glycogen phosphorylase (*pygl*), which catalyzes glycogen breakdown, 5. Glycine dehydrogenase (*gldc*), which catalyzes the reaction: glycine + H-protein-lipoyllysine → H-protein-S-aminomethyldihydrolipoyllysine + CO_2_ as part of the catabolism of glycine: 2 glycine + NAD^+^  + H_2_O → serine + CO_2_ + NH_3_ + NADH + H^+^, and 6. Dimethylarginine dimethylaminohydrolase 1 (*ddah1*), which hydrolyses methylarginine to produce dimethylamine and citrulline. DDAH-1 down regulation is associated with hypoxia.

Hearing deficits have been reported in both CHED and FECD patients^15^, which have been inadequately explained. Interestingly, decreased expression of cytoskeleton component *espn* (Espin), a microfilament binding protein mutated in autosomal dominant hearing loss^41^, was seen here. Of special interest, *ripor2* (RHO family interacting cell polarization regulator 2), encoding a membrane-associated protein of the hair cell stereocilia whose mutation causes hearing loss^42^ was among genes involved with decreased expression in *slc4a11*^*−/−*^ corneas (Table 2). Reduced expression of two genes causing genetic hearing loss is an observation worthy of additional study.

Analysis here revealed that cornea expresses 13,173 genes. Comparison to gene expression in 13 other tissues revealed the pattern of corneal expression as most similar to epithelial tissues, as expected. Also, consistent with current understanding of corneal physiology was the identification of keratin 12 (*krt12*) as the most abundantly expressed gene. *Krt12* causes Meesmann epithelial corneal dystrophy when mutated^43^. Consistent with this KRT12 has not been found to be expressed in human corneal endothelial cells^30^. Similarly, the second most abundant transcript, *col17a1*, encodes a collagen protein whose mutations cause a corneal epithelial erosion disorder^44^. The gene is, however, also amongst transcripts identified in human CEC^30^. *Piezo2*, which encodes a cation channel required for touch sensation in mice^45^, was in the top 50 most abundant corneal transcripts. This points toward a central role of piezo2 in corneal touch sensation, a critical corneal function in protecting the eye^46^. Interestingly, *PIEZO2* is among transcripts identified in human CEC suggesting a role of the channel in the endothelium^30^.

Several genes recognized as important in CEC physiology were among the most abundantly expressed. Indeed, aquaporin 3 (*aqp3*), a water channel protein, was the fifth most abundant corneal transcript. Interestingly in human corneal endothelium, AQP1 is thought to form the apical (facing aqueous humor) water conductive pathway and AQP3 is not recognized to have a role^15^. High AQP3 expression may represent a species difference between human and mice. AQP3 is however, also expressed in human CEC^30^. Notably, however, aquaporin 3 also has an important role in corneal epithelium^47^. Additionally, *col8a2* which encodes a key collagen of Descemet’s membrane and whose mutations cause some cases of FECD^48^, is the twenty-first most abundant corneal gene. *Slc4a11*, the focus of the present study, was the thirty-fourth most abundant corneal transcript and has previously been identified as among the most highly expressed CEC genes^15^. Interestingly, the RNA splicing factor gene *mbnl3*, was the fifty-third most abundant transcript and it is reported as expressed in human CEC^30^. Other mbnl isoforms are important in FECD caused by trinucleotide repeats in *TCF4*^15^, leading to the possibility that in mice mbnl3 is responsible for splicing *tcf4* transcripts.

Worthy of additional investigation is *slc14a1*, which was not only the forty-ninth most expressed gene, but also much more expressed in cornea than in comparison tissues. *Slc14a1* encodes the UT-A urea transporter that regulates cellular osmotic pressure and regulates renal urine volume and concentration^49,50^. These features hint at a potential unrecognized role of UT-A role in the cornea. Importantly, SLC14A1 was not found to be expressed in human cornea^30^ and its role is thus likely to be in corneal epithelium.

This work has two principal limitations. First, we studied mouse cornea not human cornea. This strain of *slc4a11*^*−/−*^ mice developed corneal edema and disruptions of CECs consistent with human corneal dystrophy pathology^11^. Nonetheless, this was a study of mice whose corneal biology undoubtedly differs from human. The second limitation was driven by the small size of mouse corneas: RNA was isolated from total cornea, not just the endothelial layer. Gene expression changes measured here thus reflect the cornea’s major cellular layers, epithelium and endothelium, both of which express SLC4A11^11,51^. The strongest indication that some of the expression changes seen here reflect those in the corneal epithelium are the expression of keratin isoforms and *kprp* associated with epithelium, not CEC. Comparison of the gene expression data here with human CEC expression data, pointed toward genes expressed in CEC and those more likely in epithelium or keratocytes.

Another gene expression analysis in a corneal dystrophy examined changes in normal versus FECD human corneas^29^. The 1556 mRNA abundance changes found in that study were compared to the changes seen here, revealing 13 alterations in common (Suppl. Table 3). Among the changes, only a few were the same gene but were instead different members of the same family. For example, in human FECD the keratin *KRT8* expression changed while we found that *krt4* expression changed. We consider that these differences may arise because the FECD study was in human cornea while here the changes were in mouse. Most interestingly, changes of FAM65B expression were observed in FECD cornea. We have already highlighted that this gene, also called *ripor2*, was altered in *slc4a11*^*−/−*^ mouse cornea. Together these commonalities indicate shared corneal gene expression changes in posterior corneal dystrophies.

We compared the expression profiles of corneal mRNA from adult *slc4a11*^*−/−*^ and *slc4a11*^+*/*+^ mice. Our findings revealed changes in genes involved in cytoskeletal organization, membrane association, and ECM remodeling and identified additional genes of potential importance in corneal function. Together the gene expression changes identified here capture the cornea’s response to corneal dystrophy arising from loss of *slc4a11*. Conclusions about which processes change in CEC and which arise in other corneal cell types will require future investigations.

## Materials and methods

### Animals

Animal handling was performed according to the Canadian Council on Animal Care and animal experiments were conducted in accordance with the ARVO Statement for the Use of Animals in Ophthalmic and Vision Research, with approval from the Maisonneuve-Rosemont Hospital Committee for Animal Protection. The study is reported in accordance with ARRIVE guidelines (https://arriveguidelines.org). Mice deficient for *slc4a11* have been described previously^11^.

### Isolation of mouse corneas and RNA

Adult (17 week old) male *slc4a11*^+*/*+^ and *slc4a11*^*−/−*^ mice were sacrificed and the ocular globes removed immediately. Corneas were excised outside the limbus from enucleated eyes, washed in PBS (140 mM NaCl, 3 mM KCl, 6.5 mM Na_2_HPO_4_, 1.5 mM KH_2_PO_4_, pH 7.5) and two corneas corresponding to a same animal placed into a microcentrifuge tube containing RNAlater solution (Invitrogen, Fisher Scientific). Samples were stored at -80 °C and subsequently thawed on ice before RNA extraction.

### RNA isolation and qPCR

Before RNA extraction, mouse corneas were placed in a microcentrifuge tube containing Buffer RLT Plus (from Qiagen RNeasy plus kit), and each cornea cut into 3–5 pieces with micro-dissection scissors, homogenized with a 27 gauge needle and syringe, and vortexed for 30 s before applying to gDNA Eliminator spin column. Total RNA was extracted from mouse corneas, using RNeasy Plus Qiagen RNA extraction kit (Qiagen, Ontario, CA) with effective on-column gDNA removal. The concentrations and purities of the RNA samples were analyzed on a NanoDrop spectrophotometer (Isogen, Life Science) and Bioanalyzer. All samples had an RNA integrity value > 5.0, and an A_260_: A_280_ ratio of 2.08 ± 0.03.

Total RNA (1 µg) was used to synthesize cDNA for use in conventional RT-PCR or quantitative RT-PCR (qPCR) analyses. To measure mRNA concentrations the synthesized cDNA was diluted fivefold and 2 μl of the diluted sample was used in PCR reactions with primers targeting selected genes. PCR reactions were conducted in duplicates done on three separate *slc4a11*^+*/*+^ and *slc4a11*^*−/−*^ mice (2 eyes/corneas per animal), respectively. For qPCR analyses, reactions (20 μl) contained 250 nM forward and reverse primers, 100 ng cDNA templates made from murine total RNA, and 1 × SYBR Green Supermix (Supergreen, Wisent). Thermal cycling parameters were: 95 °C for 10 min and (95 °C for 20 s, 58 °C 15 s, and 72 °C for 15 s) repeated for 40 cycles. A threshold was set at the logarithmic linear phase when it could be distinguished from the background (crossing point; denoted as Ct). Ct values for selected targets were normalized to the Ct value of glyceraldehyde 3-phosphate dehydrogenase (*gapdh*), enabling normalization according to the equation: 1/^(Ct[gene]-Ct[GAPDH])^ as an internal control. The qPCR analysis was performed using a RotorGene 3000 rapid thermal cycler system (GE Life Sciences). Suppl. Table 2 lists oligonucleotide primers and expected amplicon size used for real time qPCR analysis.

### mRNA sequencing—bioinformatics workflow

Sequencing analysis was performed at PlantBiosis, Department of Biology, University of Lethbridge.

### FastQC analysis

Initial sequencing library quality control quality control was conducted before and after adapter trimming: FastQC 0.11.8. https://www.bioinformatics.babraham.ac.uk/projects/fastqc/.

### Adapter trimming

Adapters and low-quality bases were removed using Trim Galore! Version 0.6.4 (Command trim_galore -illumina –fastqc *. fastq). https://www.bioinformatics.babraham.ac.uk/projects/trim_galore/.

### Filtering of ribosomal reads

Following ribosomal depletion procedure, remaining fractions of ribosomal reads on sequencing libraries were downloaded from the Illumina iGenome site together with GRCm38 Ensembl genome. Bowtie2 v.2.3.5.1 (Command: bowtie2 -p 20 –sensitive -x < musRibosomal_index > –un < filtered.fastq > -U < trimmed.fastq > -S < ribosomal.sam >) in ‘–sensitive’ mode was used to map trimmed reads^52^. Reads mapped to ribosomal sequences were discarded from further analysis.

### Read mapping

Trimmed and filtered reads were mapped to mouse genome (Ensembl, GRCm38) using hisat2 version 2.0.5.^31^. https://ccb.jhu.edu/software/hisat2/index.shtml (Command: hisat2 -q –rna-strandness R –phred33 -p 4 –known-splicesite < path_to_splice_site > -x < path_to_index > -U < fastq > -S < sam >). SAM files generated by hisat2 were converted to BAM, sorted and indexed using samtools 1.9^53^.

### Read counting

Reads mapping to genes were counted using featureCounts version 1.6.1 from Subread package http://bioinf.wehi.edu.au/featureCounts/ (Command: featureCounts -a < genes.gtf > -o < counts_out > -s 2 < bam1 bam2 … bamN >)^54^.

### Statistics and additional quality control using MultiQC

Reports generated by bioinformatics software used in the project were collated and presented in html format using mutiQC https://multiqc.info/.

### Exploratory analysis of gene expression in R

Exploratory analysis of gene expression included non-supervised hierarchical clustering and principal component analysis (PCA). The distances between transcriptional profiles were calculated using dist () function, and sample clustering was conducted using hclust () function from R base package. The distance measure was “Euclidean” and clustering method was “ward D2”. Sample clustering was visualized as a heatmap using pheatmap R package. Principal components analysis was conducted using prompt () function from R base.

### Clustering and principal component analysis

Briefly, raw count data was loaded into R version 3.6.1. Genes with the low expression level defined as less then 1 count/10^6^ in at least two samples were removed from the analysis. Normalization and variance stabilizing transformation was applied to raw count data as described in DESeq2 manual. Relationships between samples were explored using non-supervised hierarchical clustering and principal components analysis (PCA). The results of exploratory analysis were visualized as heatmaps and principal component plots. Clustering and PCA were based on the top 1500 of genes with the highest median absolute deviation (MAD). In the case of hierarchical clustering the distance measure was Euclidean.

### Differential expression analysis—gene ontology and Kyoto encyclopedia of genes and genomes analysis

Differentially expressed genes were detected using DESeq2 1.24.0 Bioconductor package https://bioconductor.org/packages/release/bioc/html/DESeq2.html) ^55^. Genes with adjusted p-values (Bonferroni-Hochberg adjustment for multiple comparisons) less than 0.05 (5% chance of gene being a false positive) and over 1.5-fold change in either direction were selected as differentially expressed. Kyoto Encyclopedia of Genes and Genomes (KEGG) pathway and Gene Ontology (GO) enrichment analysis was also conducted using Generally Applicable Pathway Analysis (GAGE) v.2.34.0 Bioconductor package^56^ and geneSCF v1.1-p2 command line utility^57^.

### Assessment of relative gene expression

In order to compare the relative abundance of cornea transcripts for the mice described in this study with other mouse tissues, a standardized pipeline was developed. Specifically Fastq files for this study and for E-MTAB-6081^58^ (https://www.ebi.ac.uk/ena/browser/view/ERP104395) were mapped to the latest Ensembl mouse assembly GRCm38 (ftp://ftp.ensembl.org/pub/release-87/fasta/mus_musculus/cdna/) using Kallisto^59^ and gene-level summaries generated with tximport^60^. Low expression genes were filtered out and data were converted to *log*CPM values, which were then subjected to principal component analysis. Tissue-specific expression patterns were identified from the tau metric^61^. The top 102 most highly expressed corneal-enriched transcripts were subjected to heat map analysis using normalized counts (*log*TPM) in comparison to those genes in 13 reference tissues.

### Summary of mRNA sequencing—bioinformatics workflow

RNA-Seq was performed using the Illumina NextSeq500 Platform. Library was constructed using NEBNext rRNA Depletion Kit. Transcripts were compared and referred to the Mouse GRCm38 (Ensembl) reference genome downloaded from Illumina iGENOME. Gene Ontology (GO) and the Kyoto Encyclopedia of Genes and Genomes (KEGG)^4^ were used to analyze the enrichment of transcripts.

### Statistical analysis

All of the reports generated by bioinformatics software used in the project were collated and presented in html format using mutiQC https://multiqc.info/. MultiQC results can be found in multiqc_results/directory(BernardoA_mRNA_10MAR2020/multiqc_result/multiqc_report.html). The statistical analysis of gene expression by qReal Time RT-PCR was performed using GraphPad Software, with a Student's t-test used to compare the mean of two independent groups with the difference determined to be significant if the P-value was < 0.05.

## Supplementary Information

Supplementary Information.
