# Supplementary material for: Altered gene expression in slc4a11−/− mouse cornea highlights SLC4A11 roles
Source: Sci Rep. 2021 Oct 22;11:20885. doi: 10.1038/s41598-021-98921-w (PMC8536660; doi:10.1038/s41598-021-98921-w)
Supplement: Supplementary file 1 — Supplementary Information. [file 41598_2021_98921_MOESM1_ESM.pdf]

Supplementary Table 1. Genes with significantly altered expression in *slc4a11*$^{-/-}$ mouse cornea. Genes with increased expression are written in green text and red indicates decreased expression. Genes in the table are ordered from most to least altered in both the increased and decreased categories. Fold changes indicate change of expression in *slc4a11*$^{-/-}$ mice relative to *slc4a11*$^{+/+}$. Genes with adjusted P-values (Bonferroni-Hochberg adjustment for multiple comparisons) less than 0.05 and over 1.5-fold change in either direction were selected as differentially expressed. In the gene product function column, products of differentially expressed genes are written in upper case for consistency and not in italic. * indicates genes not identified as expressed in human CEC (on the basis of genes expressed in isolated human CEC[1,2]) and thus likely expressed in corneal epithelial cells. One of these studies measured transcript abundance in 15 human CEC samples[2]. In this case, we considered genes as expressed in CEC if they were found in 10/15 samples.

Entrez_id/ gene	Adjusted P-value	*log*$_2$ Fold Change	Fold Change	Gene Product Function
14394/ *gabra1*	*8.5×10^{-6}	4.1	18	• Gamma-aminobutyric acid receptor (GABAR)[3].<br>• GABA is an inhibitory neurotransmitter causing Cl^- influx cell hyperpolarization.
14401/ *gabrb2*	*3.0×10^{-4}	2.8	6.7	• GABA receptor subunit[4].
NA/ *gv2c*	*3.6×10^{-9}	2.3	4.8	• Synaptic vesicle glycoprotein 2[5].<br>• Involved in Ca^{2+} dependent synaptic vesicle exocytosis.
NA/ *kcnj13*	*4.0×10^{-4}	2.0	4.1	• Inwardly rectifying K^+ channel[6].
70945/ *mmrn1*	*2.1×10^{-2}	2.0	4.0	• Multimerin (MMRN) is involved in endothelial cell adhesion.<br>• Aids in extracellular matrix elasticity.<br>•Binds outer surface of endothelial cells and extracellular matrix (ECM) (including collagen) to aid in adhesion[7].
14201/ *fhl3*	6.0×10^{-3}	1.9	3.8	• Four-and-a-half LIM domains 3 (FHL3) inhibits regulates epithelial-mesenchymal transition (EMT)[8].<br>• Regulator of myoblast differentiation- acts as a transcriptional activator or repressor[9].
102954/ *nudt11*	2.5×10^{-2}	1.9	3.8	• Nucleoside diphosphate attached moiety 'X'-type (NUDT) is a phosphohydrolase that hydrolyses 5-phosphoribosyl 1-pyrophosphate (PRPP) to generate glycolytic activator, ribose 1,5 bisphosphate[10].

66042/ *sostdc1*	*7.0×10^{-4}	1.9	3.8	• Sclerostin domain containing 1 (SOSTDC1) is a bone morphogenetic protein (BMP) antagonist and Wnt pathway antagonist, regulating BMP signaling during cellular proliferation, differentiation and programmed cell death[11,12].
226075/ *glis3*	6.6×10^{-16}	1.9	3.7	• GLI-Similar protein 3 (GLIS3) is a Krüppel-like zinc finger transcription factor[13,14].<br>• Regulator of embryonic development and cell migration.
243931/ *tshz3*	3.4×10^{-2}	1.9	3.6	• Teashirt zinc finger homeobox family member 3 (TSHZ3; also known as ZNF537) encodes a zinc finger transcription factor upregulated upon oxidative damage[15,16].<br>• TSHZ3 overexpression in myoblasts inhibited myogenic differentiation and decreased Myog, a transcription factor that induces myogenesis, expression.
NA/ *nt5e*	1.53×10^{-7}	1.8	3.6	• Ecto-5'-nucleotidase (NT5E) catalyzes the conversion of extracellular nucleotides to nucleosides.<br>• The preferred substrate of the enzyme is AMP[17].
12296/ *cacnb2*	1.5×10^{-2}	1.8	3.5	• CACNb2 is the β2 subunit of the L-type calcium channel, involved in positive regulation of presynaptic cytosolic calcium levels[18].
319211/ *nol4*	1.7×10^{-6}	1.8	3.5	• Nucleolar protein 4 (NOL4) is involved in determining cell specificity.<br>• Abundant in malignant cells[19].
72432/ *spink5*	5.2×10^{-7}	1.8	3.5	• *Serine peptidase inhibitor* Kazal type 5 (*SPINK5*) is associated with dermatitis and asthma[20-22].
22153/ *tubb4a*	1.5×10^{-3}	1.7	3.3	• *TUBB4A* encodes beta-tubulin isotype, beta-tubulin 4A, a core tubulin protein that forms microtubules, a structural constituent of the cytoskeleton[23,24].
21928/ *tnfaip2*	9.6×10^{-3}	1.7	3.3	• Tumor necrosis factor alpha induced protein (TNFAIP*) is* Involved in cell determination and organ formation.<br>• Involved in regulating inflammation and apoptosis of tumor cells[25].
117600/ *srgap1*	2.3×10^{-10}	1.7	3.3	• Slit-Robo GTPase-activating protein 1 (SRGAP1) negatively regulates neuronal migration by regulating Rac activity.<br>• SRGAP1 limits Rac1 activity, decreasing cell migration[26,27].
242122/ *vtcn1*	*4.9×10^{-6}	1.7	3.2	• VTCN1 (V-set domain containing T-cell activation inhibitor 1) proteins are present on the surface of antigen-presenting cells and interact with ligand bound to receptors on the surface of T cells.

				• Decreases cytokine production and decreases T-cell proliferation[28].
16980/ *lrrn2*	3.7 x 10^{-2}	1.6	3.1	• Leucine rich repeat neuronal 2(LRRN2) LrRRN2 is a cell adhesion molecule and signal transduction receptor with a type III fibronectin domain in its extracellular domain.<br>• Lrrn2 possesses ww domains in the short intracellular region- these mediate protein–protein interactions[29].
243312/ *elfn1*	*6.0 x 10^{-3}	1.6	3.1	• Extracellular leucine rich repeat and fibronectin Type III domain containing 1. (ELFN1) is important in synapse formation, neuronal adhesion and synapse differentiation by recruiting pre/postsynaptic proteins[30,31].
16682/ *krt4*	*1.8 x 10^{-8}	1.6	3.0	• Keratin 4 (KRT4) forms intermediate filaments, a structural component of the cytoskeleton in all epithelial cells[32].
14086/ *fscn1*	1.6 x 10^{-4}	1.6	3.0	• Fascin (FSCN1) is an actin binding and bundling protein that directly interacts with to control focal adhesions.<br>• Fascin is also required for the stability of parallel bundles of F-actin.<br>• Fascin–actin binding plays a role in regulating cell adhesion[33].
13380/ *dkk1*	3.7 x 10^{-7}	1.5	2.9	• Dickkopf-related protein 1 (DKK1) is a Wnt pathway antagonist.<br>• A regulator in local cell adhesion, Dkk1 localizes to the plasma membrane, to adhesion complexes, and regions of high actomyosin.<br>• It is a repressor of cell polarization and cell-cell adhesion.<br>• Prevents the localization of β-catenin[34].
106565/ *dlk2*	3.6 x 10^{-6}	1.5	2.8	• Delta-like 2 (DLK2) is a negative regulator of Notch signaling pathway.<br>• Notch signaling plays an important role in proliferation[35].
12818/ *col14a1*	4.1 x 10^{-2}	1.5	2.7	• *COL14a1* encodes the alpha chain of type XIV collagen, a component of ECM.<br>• *col14a1*$^{-/-}$ mice displayed delayed corneal endothelial maturation, had thicker cornea suggesting that collagen XIV plays a role in corneal thinning needed to maintain corneal clarity and also had delayed endothelial junction maturation [36].
107146/ *glyat*	*3.0 x 10^{-2}	1.4	2.7	• Glycine-N-acyltransferase (GLYAT) plays a role in conjugating glycine with mitochondrial acyl-CoA.<br>• Reduced expression of GLYAT causes hyperglycemia meaning it is an important regulator in energy metabolism.<br>• Overexpression of GLYAT decreases glucose levels[37].

225288/ *fhod3*	1.5×10^{-8}	1.4	2.6	• Formin Homology 2 Domain Containing 3 (FHOD3) is an actin associated protein that plays a role in actin organization.<br>• Formins direct the formation of straight actin filaments such as stress fibers[38].
11839/ areg	3.0×10^{-3}	1.4	2.6	• Amphiregulin (AREG) binds to and regulates epidermal growth factor receptor (EGFR) and acts as an autocrine growth factor to promote epithelial cell growth.<br>• Important in the regulation of the EMT in pancreatic cancer.<br>• Silencing of AREG causes keratinocyte growth arrest[39,40].
16664/ *krt14*	*1.1×10^{-26}	1.3	2.5	• Keratin 14 (KRT14) is a constituent of the cytoskeleton, forming part of intermediate filaments[41-43].
17918/ *myo5a*	4.2×10^{-8}	1.3	2.5	• Myosin gene superfamily member 5A (MYO5A) is one of the three myosin V heavy-chain genes, part of an actin-based motor protein.<br>• Myo5a can dissociate from the ribosome complex to release ribosomes from the actin cytoskeleton[44].
50918/ *myadm*	8.4×10^{-12}	1.3	2.5	• Myeloid associated differentiation marker (MYADM) is a transmembrane protein at the plasma membrane where it co-localizes with actin and cell-cell junctions.<br>• When MYADM was silenced it caused disruption of the endothelial monolayer and caused disorganization of cell-cell junctions.<br>• MYADM organizes plasma membrane domains and regulates the connection with cytoskeleton[45,46].
18599/ *padi1*	*2.5×10^{-2}	1.3	2.5	• Peptidylarginine deiminases (PADIs) catalyze deamination of proteins by converting arginine into citrulline.<br>• Histone citrullination turns off gene expression and is an antagonist to histone arginine methylation[47,48].
17755/ *map1b*	4.8×10^{-11}	1.3	2.4	• Microtubule associated protein 1b (MAP1B) is important in microtubule assembly[49].
20677/ sox4	1.1×10^{-3}	1.3	2.4	• SRY-box transcription factor 4 (SOX4) is involved in the regulation of embryonic development and determination of cell fate.<br>• Functions in the apoptosis pathway[50].
16665/ *krt15*	1.6×10^{-26}	1.3	2.4	• Keratins, like keratin 15 (KRT15) are intermediate filament proteins that are responsible for the structural integrity of epithelial cells.
19228/ *pth1r*	2.0×10^{-3}	1.3	2.4	• PTH type 1 receptor (PTH1R) is a G-protein coupled receptor involved in calcium homeostasis.

				• PTH1R activation increases cAMP levels via adenylate cyclase activity[51].
19224/ *ptgs1*	6.6 x 10^{-16}	1.3	2.4	• Cyclo-oxygenase 1 (COX-1) (encoded by *PTGS1*) converts arachidonic acid into prostaglandins[52].
53416/ *stk39*	6.0 x 10^{-5}	1.3	2.4	• STE20 (sterile 20-like)-related proline-alanine-rich kinase (SPAK) is encoded by *STK39*.<br>• STE20 kinases are important in osmotic stress signaling.<br>•SPAK is ubiquitously expressed and modulates ion and fluid homeostasis through phosphorylation of chloride transporters and p38 MAPK pathway activation[53].
12560/ *cdh3*	4.0 x 10^{-8}	1.2	2.3	• Cadherin3 (CDH3) is a calcium-dependent cell-cell adhesion protein[54].
NA/ *krt90*	*1.0 x 10^{-3}	1.2	2.3	• Member of the keratin family of intermediate filament proteins.
12554/ *cdh13*	2.5 x 10^{-10}	1.2	2.3	• Cadherin13 (CDH13) is a member of cadherin family of cell adhesion proteins.[55].
94352/ *loxl2*	5.5 x 10^{-6}	1.2	2.3	• Lysyl oxidase 2 (LOXl2) is an extracellular amine oxidase that catalyzes the formation of crosslinks between collagen and elastin to remodel ECM.<br>• LOXL2 mediated deamination of tropoelastin increases resistance to degradation and stiffness of elastin[56,57].
13131/ *dab1*	*3.9 x 10^{-5}	1.2	2.3	• Disabled 1 (DAB1) is an intracellular adaptor protein of the Reelin signaling pathway<br>• Causes a signaling cascade of actin and microtubules.<br>• If DAB1 is phosphorylated, it activates the Crk pathway, which plays an important role in cell adhesion to fibronectin and regulates nectins and N-cadherin trafficking, important in cell-cell interactions[58].
19224/ *ptges*	6.6 x 10^{-16}	1.2	2.2	• Prostaglandin E Synthase (PTGES) is downstream of COX-2 in the prostaglandin signaling pathway.<br>• PTGES is induced by hypoxic conditions.<br>• PTGES contributes to collagen induced arthritis as determined by knockout mice studies[59,60].
16858/ *lgals7*	*1.3 x 10^{-11}	1.1	2.2	• Galectins (encoded by LGALS genes) modulate cell-cell and cell-matrix interactions.<br>• Galectin-7 binds to the extracellular domain of E-cadherin[61,62].

76898/ *b3gat1*	*6.0 x 10^{-3}	1.1	2.2	• β-1,3-glucuronyltransferase 1 (B3GAT1), a key enzyme in the glucuronyl transfer reaction is important in integrin signaling and cell motility[63,64].
104111/ *adcy3*	3.6 x 10^{-4}	1.1	2.2	• ADCY3 is an adenylate cyclase that catalyzes the synthesis of cAMP from ATP.<br>• ADCY3 has a role in metabolism and regulation of glucose homeostasis and protects from type 2 diabetes when it is upregulated[65].
22271/ *upp1*	1.8 x 10^{-14}	1.1	2.1	• Uridine phosphorylase 1 (UPP1) catalyzes the phosphorylation of uridine to uracil.<br>• Plays an important role in uridine homeostasis[66].
55963/ *slc1a4*	1.6 x 10^{-08}	1.1	2.1	• Solute carrier family 1 member 4 (SLC1a4) is a sodium-dependent neutral amino acid transporter that transports glutamine into the cell to be used for glutaminolysis[67,68].
NA/ *krt19*	2.0 x 10^{-10}	1.1	2.1	• Part of the keratin family which are intermediate filaments involved in maintaining structural integrity of epithelial cells.<br>• Interact with various proteins and effector molecules to regulate cell growth, migration and apoptosis[69,70].
17181/ *matn2*	8.7 x 10^{-5}	1.0	2.1	• Matrilin2 (MATN2) is important in ECM organization.<br>• Interacts with ECM molecules using a von Willebrand Factor A- like domain.<br>• Forms ECM filaments by interacting with itself or other ECM constituents, including collagens and fibronectin.<br>• Matrillin2 binds to cells using α1β1 integrin[71].
240888/ *gpr161*	1.6 x 10^{-2}	1.0	2.0	• A G-protein coupled receptor (GPCR) that increases intracellular cAMP levels.<br>• Suppresses Shh signaling.<br>• Shh signaling is important in embryonic cell differentiation[72].
68655/ *fndc1*	2.5 x 10^{-13}	1.0	2.0	• Fibronectin type III domain containing 1 (FNDC1) is a major structural domain of fibronectin, part of ECM.<br>• Fndc1 expression levels may be an important biomarker for gastric cancer and is involved in regulating tumorigenesis[73].
76448/ *ppp1r18*	2.5 x 10^{-2}	1.0	2.0	• Protein phosphatase 1 regulatory subunit 18 (PPP1r18) overexpression negatively regulates actin ring formation.<br>• Suppresses actin elongation by directly binding to actin[74].
319757/ *smo*	1.4 x 10^{-9}	1.0	2.0	• Smoothened (SMO) is a G protein-coupled receptor involved with patched proteins in the Hedgehog signaling pathway located on the plasma membrane where Hh signal is transduced.

				• SMO activation and activation of Cubitus interruptus (Ci)/Gli family of zinc finger transcription factors which then causes expression of Hh target genes[75].
208439/ *klhl29*	1.0×10^{-3}	1.0	2.0	• Kelch-like (*KLHL*) proteins are substrate-specific adapters for cullin E3 ubiquitination[76]. There is no literature on member 29.
237175/ adgrg2	$*2.0 \times 10^{-2}$	1.0	2.0	• Adhesion G-protein coupled receptor G2 (ADGRG2) is a member of the adhesion family of GPCRs involved in cell-cell communication and cell-matrix interaction[77-79].
108043/ *chrnb3*	$*1.0 \times 10^{-11}$	-4.9	0.03	• Cholinergic receptor nicotinic beta 3 subunit (Chrnb3) is part of a superfamily of ligand-gated ion channels at synapses that mediate neuronal signals[80,81].
21892/ *tll1*	$*7.4 \times 10^{-6}$	-3.3	0.10	• Tolloid-like protein 1 (TLL1) negatively regulates hepatic differentiation of human iPS cells via upregulation of TGFβ signaling.<br>• Down regulation of Tll1 increased cell differentiation[82].
329872/ *frem1*	2.4×10^{-14}	-3.2	0.11	• FRAS1-related extracellular matrix 1 (FREM1) controls adhesion of the developing epidermis.<br>• FREM1 mutation contributes to basement membrane fragility and blistering maybe by reduced PDGFC signaling leading to impaired ECM function.<br>• FREM1 may bind directly to growth factor A (PDGFA), fibroblast growth factor FGF2, and collagens V and VI[83,84].
238377/ *gpr68*	1.9×10^{-5}	-3.0	0.12	• G-protein-coupled receptor 68 gene (GPR68) is an acid-sensing receptor[85].<br>• GPR68 requires the presence of protons to sense stress to activate receptor[86,87].
13081/ *cyp24a1*	1.4×10^{-6}	-3.0	0.12	• Member of the cytochrome P450 superfamily of enzymes.<br>• Enzymatic pathway for cholesterol, steroids, and lipids[22,88,89].
20452/ *st8sia4*	3.8×10^{-4}	-2.2	0.22	• ST8SIA4 is a polysialyltransferase that catalyzes polycondensation of alpha-2,8-linked sialic acid.<br>• ST8SIA4 delays oligodendrocyte differentiation, and plays a role in re-myelination[90,91].
53417/ *hif3a*	1.7×10^{-2}	-2.1	0.24	• Hypoxia inducible Factor 3 Alpha Subunit (HIF3A) is involved in response to hypoxia, including transition to anaerobic metabolism.<br>• Promotes glucose uptake by activating the transcription of transporters GLUT1 and GLUT3.

				• Inhibits PDH (pyruvate dehydrogenase) complex and blocks conversion of pyruvate into acetyl-CoA[92].
333315/ *frem3*	*7.5 x 10^{-39}	-2.0	0.25	• FRAS-related extracellular matrix 3 (FREM3) is an ECM protein associated with epithelial–mesenchymal cohesion during embryonic development in mammals. • FREM3 is an integral membrane protein containing numerous CSPG (chondroitin sulfate proteoglycan element) repeats and Calx-beta domains[83,84].
56226/ *espn*	5.7 x 10^{-3}	-2.0	0.25	• Espin (ESPN) is a multifunctional actin-bundling protein. With a major role in regulating the organization, dimensions, dynamics, and signaling capacities of the actin filament rich. • ESPN interacts with F-acting to mediate the formation and extension of protrusion structure[93].
13395/ *dlx5*	*2.4 x 10^{-2}	-1.9	0.28	• Distal-Less Homeobox 5 (DLX5) is a transcription factor with a role in bone formation, neurogenesis and hematopoiesis as part of the bone morphogenetic protein (BMP) signaling pathway. • In ovarian cancer, DLX5 promotes cell proliferation by increased stimulation of AKT signaling by transactivation of insulin receptor substrate 2[94].
18208/ *ntn1*	1.0 x 10^{-15}	-1.8	0.29	• Netrin-1 (NTN1) is an axonal guidance molecule that also promotes angiogenesis. NTN1 upregulation inhibits apoptosis induced via its dependence and possible cross-linking to on DCC (deleted in colorectal cancer (DCC) protein) and UNC5H. • NTN1 is upregulated in in Type 2 brittle cornea syndrome (BCS2) studying mutations in the transcription factor PR domain containing 5 (PRDM5). • Dysregulated PRDM5-target genes reveal enrichment for extracellular matrix (ECM) genes supporting vascular integrity and development[95-98].
110095/ *pygl*	6.4 x 10^{-37}	-1.8	0.29	• Glycogen phosphorylase L (PYGL) catalyses breakdown of glycogen to glucose. • Mutations can cause liver phosphorylase deficiency, which may result in glycogenolysis disorder like Hers Disease[99,100].
26971/ *pla2g2f*	*3.3 x 10^{-2}	-1.8	0.29	• Phospholipase 2g2f (PLA2G2F) is a secreted enzyme hydrolyzing phospholipids into free fatty acids and lysophospholipids, with a role in epidermal barrier function[101]. • In humans and mice, PLA2G2F causes release of arachidonic acid, interleukin-1 and microsomal prostaglandin E synthase.

171531/ *mlph*	2.5 x 10^{-9}	-1.7	0.31	• Melanophilin (*MLPH*) is a critical component of the melanosome transport machinery and may be part of a transport complex with Rab27a and MyoVa[102,103].
108017/ *fxyd4*	6.0 x 10^{-4}	-1.6	0.32	• FXYD4 is a member of a family of Na^+-K^+-ATPase stimulators[104,105].
76074/ *gbp8*	*3.2 x 10^{-2}	-1.6	0.33	• Guanylate binding proteins (GBPs) are GTPases up-regulated by proinflammatory cytokines and toll-like receptor agonists[106,107].
252838/ *tox*	9.9 x 10^{-12}	-1.6	0.33	• Thymocyte Selection-Associated High Mobility Group Box (TOX) is a transcription factor with uncharacterized roles in the nervous system.<br>• TOX is regulated by calcineurin/Nfat signalling[108].
18383/ *tnfrsf11b*	7.7 x 10^{-6}	-1.6	0.33	• Tumor necrosis factor receptor superfamily member 11B (*TNFRSF11B*) encodes osteoprotegerin, a decoy receptor for receptor activator of NF-κB ligand (RANKL)[109,110].
83672/ *sytl3*	1.1 x 10^{-3}	-1.6	0.34	• Synaptotagmin (Syt)-like protein (Slp) (SYTL) is involved in vesicle trafficking[111].
433619/ *kprp*	*1.0 x 10^{-2}	-1.5	0.35	• Keratinocyte Proline-Rich Protein (KPRP) is involved in keratinocyte differentiation.<br>• KPRP is expressed exclusively in stratified squamous epithelial layers[112].
12268/ *c4b*	4.5 x 10^{-2}	-1.5	0.35	• Complement component 4 (C4) is a part of host defense against microbes.<br>• Patients deficient in this protein have compromised immunity[113].
234356/ *csgalnact1*	1.5 x 10^{-13}	-1.5	0.36	• Chondroitin sulfate N-acetylgalactosaminyltransferase-1 (CSGalNAcT1) is involved in chondroitin sulfate lengthening in proteoglycans.<br>• Required for normal cartilage development, brain development, mechanical properties of connective tissue, and aggrecan metabolism.<br>• Component of the extracellular matrix[114,115].
219134/ *shisa2*	3.9 x 10^{-5}	-1.5	0.36	• Single transmembrane segment protein acting as a protein-protein interaction partner, regulating signaling pathways.<br>• Involved in downregulation of pathways of fibroblast growth[116].

381310/ stum	**3.3 x 10^{-2}*	-1.4	0.37	• *Drosophila* mutants of *STUM* stumble, hence the gene name. Gene may be responsible for the generation of the proper proprioceptive responses in stum-expressing neurons<br>• Essential for converting cell stretch of dendrites into cellular responses[117].
407795/ *smim31*	*1.1 x 10^{-5}	-1.4	0.39	• Small integral membrane protein 31 (SMIM31) promoted cell proliferation and repressed cell apoptosis in head and neck squamous cell carcinoma cells[118].
12804/ *cntfr*	*3.1 x 10^{-2}	-1.4	0.39	• Ciliary neurotrophic factor receptor (CNTFR) promotes differentiation and survival of a wide range of nervous cells. Also promotes motor neurons viability by preventing their degeneration[119].
70809/ *clec2g*	*6.2 x 10^{-6}	-1.3	0.39	• C-type lectin 2G (SLEG2G) has been proposed to have a role in epithelial stratification or cornification[120,121].
68339/ *ccdc88c*	3.9 x 10^{-2}	-1.3	0.40	• CCDC88C encodes DAPLE, a regulator of epithelial cell polarity[122,123].
239559/ *a4galt*	1.5 x 10^{-5}	-1.3	0.40	• α-1,4-galactosyltransferase (A4GALT) is required for globotriaosylceramide (GB3) synthesis. GB3 is found on the surface of epithelial and endothelial cells where it acts as a protein receptor[124,125].
22361/ *vnn1*	*1.6 x 10^{-12}	-1.2	0.43	• Vanin-1 (VNN1) is anchored to the cell surface by glycosylphosphatidylinositol and hydrolyzes pantetheine to pantothenic acid and cysteamine.<br>• In oxidative phosphorylation, Vnn1 permits restoration of CoA pools thus controlling ATP production.<br>• VNN1 limits the Warburg effect and promotes mitochondrial function[126,127].
56808/ *cacna2d2*	3.5 x 10^{-2}	-1.2	0.44	• Voltage-gated calcium channel auxiliary subunit alpha2delta 2 gene (CACNA2D2) controls calcium currents[128].
329628/ *fat4*	7.3 x 10^{-3}	-1.2	0.44	• FAT is a protocadherin that binds the protein, DCHS, on adjacent cells to regulate planar cell polarity[129].
NA/ *lce3a*	*3.5 x 10^{-3}	-1.2	0.45	• Late cornified envelope 3a (LCE3A) is involved in keratinization and skin repair.<br>• Loss of LCE3 function may compromise ability of psoriatic lesions to heal[130].
104174/ *gldc*	4.4 x 10^{-17}	-1.2	0.45	• Glycine dehydrogenase (GLDC) decarboxylates glycine and moves a one-carbon unit into folate metabolism.<br>• GLDC reduction causes abnormal tissue folate profiles, slow

				growth, and lessened cellular proliferation<br>• GLDC pathways include glyoxylate metabolism and glycine degradation[131].
193385/ *ripor2*	3.8 x 10^{-6}	-1.1	0.46	• RHO family interacting cell polarization regulator 2 (RIPOR2, previously known as FAM65B) is an essential protein required in human hearing, that acts as an inhibitor of the small G protein RhoA[132].<br>• Regulates abundance and post translational modifications of proteins expressed in the kinocilium.
NA/ *ccn3*	*4.5 x 10^{-3}	-1.1	0.46	• Cellular communication network factor 3 (CCN3) interacts with extracellular and transmembrane proteins to regulate expression of ECM genes[133,134].
100129/ *gpr153*	2.2 x 10^{-4}	-1.1	0.47	• G-protein coupled receptor primarily expressed in CNS.<br>• Mutations are associated with neuropsychiatric diseases (schizophrenia, autism).<br>• Related to serotonin receptors that are involved in body weight homeostasis[135].
192198/ *lrrc4*	1.6 x 10^{-5}	-1.1	0.47	• LRRC4/NGL-2 (Leucine rich repeat containing 4/Netrin-G ligand-2) is important in neural development and glioma formation.<br>• LRRC4 inhibits glioblastoma cell proliferation by downregulating pleiotropic cytokines[136,137].
77462/ *tmem116*	1.7 x 10^{-2}	-1.1	0.47	• TMEM116 is a transmembrane protein of unknown function.
235320/ *zbtb16*	2.1 x 10^{-5}	-1.1	0.48	• ZBTB16 is a zinc-finger family transcription factor[138].
68939/ *rasl11b*	1.2 x 10^{-4}	-1.0	0.49	• Ras-like protein family member 11B (RASL11B) is a GTPase.<br>• Upregulated RASL11B inhibits cell proliferation, invasion, and migration; induce G0/G1 cell cycle arrest and promotes cell[139].
69219/ *ddah1*	8.7 x 10^{-11}	-1.0	0.50	• Dimethylarginine dimethylaminohydrolase 1 (DDAH-1) hydrolyses methylarginine to produce dimethylamine and citrulline. Its down regulation is associated with hypoxia and its inhibition blocks NO snthesis[140-142].

References

1 Bohnsack, J. P., Patel, V. K. & Morrow, A. L. Ethanol Exposure Regulates Gabra1 Expression via Histone Deacetylation at the Promoter in Cultured Cortical Neurons. *J Pharmacol Exp Ther* **363**, 1-11, doi:10.1124/jpet.117.242446 (2017).

2 Zhang, T. *et al.* Meta-analysis of GABRB2 polymorphisms and the risk of schizophrenia combined with GWAS data of the Han Chinese population and psychiatric genomics consortium. *PLoS One* **13**, e0198690, doi:10.1371/journal.pone.0198690 (2018).

3 Berger, T. C. *et al.* Neuronal and glial DNA methylation and gene expression changes in early epileptogenesis. *PLoS One* **14**, e0226575, doi:10.1371/journal.pone.0226575 (2019).

4 Roman, D., Zhong, H., Yaklichkin, S., Chen, R. & Mardon, G. Conditional loss of Kcnj13 in the retinal pigment epithelium causes photoreceptor degeneration. *Exp Eye Res* **176**, 219-226, doi:10.1016/j.exer.2018.07.014 (2018).

5 Parker, D. N. *et al.* The functions of the A1A2A3 domains in von Willebrand factor include multimerin 1 binding. *Thromb Haemost* **116**, 87-95, doi:10.1160/TH15-09-0700 (2016).

6 Li, P. *et al.* FHL3 promotes pancreatic cancer invasion and metastasis through preventing the ubiquitination degradation of EMT associated transcription factors. *Aging (Albany NY)* **12**, 53-69, doi:10.18632/aging.102564 (2020).

7 Zhang, Y. *et al.* FHL3 differentially regulates the expression of MyHC isoforms through interactions with MyoD and pCREB. *Cell Signal* **28**, 60-73, doi:10.1016/j.cellsig.2015.10.008 (2016).

8 Hua, L. V., Hidaka, K., Pesesse, X., Barnes, L. D. & Shears, S. B. Paralogous murine Nudt10 and Nudt11 genes have differential expression patterns but encode identical proteins that are physiologically competent diphosphoinositol polyphosphate phosphohydrolases. *Biochem J* **373**, 81-89, doi:10.1042/BJ20030142 (2003).

9 Valkenburg, K. C., Graveel, C. R., Zylstra-Diegel, C. R., Zhong, Z. & Williams, B. O. Wnt/beta-catenin Signaling in Normal and Cancer Stem Cells. *Cancers (Basel)* **3**, 2050-2079, doi:10.3390/cancers3022050 (2011).

10 Collette, N. M. *et al.* Sost and its paralog Sostdc1 coordinate digit number in a Gli3-dependent manner. *Dev Biol* **383**, 90-105, doi:10.1016/j.ydbio.2013.08.015 (2013).

11 Kim, Y. S., Nakanishi, G., Lewandoski, M. & Jetten, A. M. GLIS3, a novel member of the GLIS subfamily of Kruppel-like zinc finger proteins with repressor and activation functions. *Nucleic Acids Res* **31**, 5513-5525, doi:10.1093/nar/gkg776 (2003).

12 Jetten, A. M. GLIS1-3 transcription factors: critical roles in the regulation of multiple physiological processes and diseases. *Cell Mol Life Sci* **75**, 3473-3494, doi:10.1007/s00018-018-2841-9 (2018).

13 Faralli, H. *et al.* Teashirt-3, a novel regulator of muscle differentiation, associates with BRG1-associated factor 57 (BAF57) to inhibit myogenin gene expression. *J Biol Chem* **286**, 23498-23510, doi:10.1074/jbc.M110.206003 (2011).

14 Meng, X., Zhu, Y., Tao, L., Zhao, S. & Qiu, S. MicroRNA-125b-1-3p mediates intervertebral disc degeneration in rats by targeting teashirt zinc finger homeobox 3. *Exp Ther Med* **15**, 2627-2633, doi:10.3892/etm.2018.5715 (2018).

15 Kordass, T., Osen, W. & Eichmuller, S. B. Controlling the Immune Suppressor: Transcription Factors and MicroRNAs Regulating CD73/NT5E. *Front Immunol* **9**, 813, doi:10.3389/fimmu.2018.00813 (2018).
16 Viard, P. *et al.* PI3K promotes voltage-dependent calcium channel trafficking to the plasma membrane. *Nat Neurosci* **7**, 939-946, doi:10.1038/nn1300 (2004).
17 Zasada, M. *et al.* Short- and long-term impact of hyperoxia on the blood and retinal cells' transcriptome in a mouse model of oxygen-induced retinopathy. *Pediatr Res* **87**, 485-493, doi:10.1038/s41390-019-0598-y (2020).
18 Bitoun, E. *et al.* Netherton syndrome: disease expression and spectrum of SPINK5 mutations in 21 families. *J Invest Dermatol* **118**, 352-361, doi:10.1046/j.1523-1747.2002.01603.x (2002).
19 Chavanas, S. *et al.* Mutations in SPINK5, encoding a serine protease inhibitor, cause Netherton syndrome. *Nat Genet* **25**, 141-142, doi:10.1038/75977 (2000).
20 Veldurthy, V. *et al.* Vitamin D, calcium homeostasis and aging. *Bone Res* **4**, 16041, doi:10.1038/boneres.2016.41 (2016).
21 Cushion, T. D. *et al.* De novo mutations in the beta-tubulin gene TUBB2A cause simplified gyral patterning and infantile-onset epilepsy. *Am J Hum Genet* **94**, 634-641, doi:10.1016/j.ajhg.2014.03.009 (2014).
22 Xiao, J., Vemula, S. R. & LeDoux, M. S. Recent advances in the genetics of dystonia. *Curr Neurol Neurosci Rep* **14**, 462, doi:10.1007/s11910-014-0462-8 (2014).
23 Guo, F. *et al.* Correlation Between TNFAIP2 Gene Polymorphism and Prediction/Prognosis for Gastric Cancer and Its Effect on TNFAIP2 Protein Expression. *Front Oncol* **10**, 1127, doi:10.3389/fonc.2020.01127 (2020).
24 Liang, X., Kiru, S., Gomez, G. A. & Yap, A. S. Regulated recruitment of SRGAP1 modulates RhoA signaling for contractility during epithelial junction maturation. *Cytoskeleton (Hoboken)* **75**, 61-69, doi:10.1002/cm.21420 (2018).
25 Yamazaki, D., Itoh, T., Miki, H. & Takenawa, T. srGAP1 regulates lamellipodial dynamics and cell migratory behavior by modulating Rac1 activity. *Mol Biol Cell* **24**, 3393-3405, doi:10.1091/mbc.E13-04-0178 (2013).
26 Radichev, I. A. *et al.* Nardilysin-dependent proteolysis of cell-associated VTCN1 (B7-H4) marks type 1 diabetes development. *Diabetes* **63**, 3470-3482, doi:10.2337/db14-0213 (2014).
27 Sheikh, A. *et al.* Unfavorable neuroblastoma prognostic factor NLRR2 inhibits cell differentiation by transcriptional induction through JNK pathway. *Cancer Sci* **107**, 1223-1232, doi:10.1111/cas.13003 (2016).
28 Dunn, H. A., Patil, D. N., Cao, Y., Orlandi, C. & Martemyanov, K. A. Synaptic adhesion protein ELFN1 is a selective allosteric modulator of group III metabotropic glutamate receptors in trans. *Proc Natl Acad Sci U S A* **115**, 5022-5027, doi:10.1073/pnas.1722498115 (2018).
29 Park, D. H. *et al.* N-linked glycosylation of the mGlu7 receptor regulates the forward trafficking and transsynaptic interaction with Elfn1. *Faseb j* **34**, 14977-14996, doi:10.1096/fj.202001544R (2020).
30 Kurklu, E. *et al.* Clinical features and molecular genetic analysis in a Turkish family with oral white sponge nevus. *Med Oral Patol Oral Cir Bucal* **23**, e144-e150, doi:10.4317/medoral.21437 (2018).

31 Villari, G. *et al.* A direct interaction between fascin and microtubules contributes to adhesion dynamics and cell migration. *J Cell Sci* **128**, 4601-4614, doi:10.1242/jcs.175760 (2015).
32 Johansson, M., Giger, F. A., Fielding, T. & Houart, C. Dkk1 Controls Cell-Cell Interaction through Regulation of Non-nuclear beta-Catenin Pools. *Dev Cell* **51**, 775-786 e773, doi:10.1016/j.devcel.2019.10.026 (2019).
33 Vegh, A. *et al.* Comprehensive Analysis of DWARF14-LIKE2 (DLK2) Reveals Its Functional Divergence from Strigolactone-Related Paralogs. *Front Plant Sci* **8**, 1641, doi:10.3389/fpls.2017.01641 (2017).
34 Hemmavanh, C., Koch, M., Birk, D. E. & Espana, E. M. Abnormal corneal endothelial maturation in collagen XII and XIV null mice. *Invest Ophthalmol Vis Sci* **54**, 3297-3308, doi:10.1167/iovs.12-11456 (2013).
35 Guo, Y. F. *et al.* Suggestion of GLYAT gene underlying variation of bone size and body lean mass as revealed by a bivariate genome-wide association study. *Hum Genet* **132**, 189-199, doi:10.1007/s00439-012-1236-5 (2013).
36 Matsuyama, S. *et al.* Interaction between cardiac myosin-binding protein C and formin Fhod3. *Proc Natl Acad Sci U S A* **115**, E4386-E4395, doi:10.1073/pnas.1716498115 (2018).
37 Wang, L. *et al.* AREG mediates the epithelialmesenchymal transition in pancreatic cancer cells via the EGFR/ERK/NFkappaB signalling pathway. *Oncol Rep* **43**, 1558-1568, doi:10.3892/or.2020.7523 (2020).
38 Stoll, S. W. *et al.* Membrane-Tethered Intracellular Domain of Amphiregulin Promotes Keratinocyte Proliferation. *J Invest Dermatol* **136**, 444-452, doi:10.1016/j.jid.2015.10.061 (2016).
39 Lin, Z. *et al.* Stabilizing mutations of KLHL24 ubiquitin ligase cause loss of keratin 14 and human skin fragility. *Nat Genet* **48**, 1508-1516, doi:10.1038/ng.3701 (2016).
40 Suresh, B., Lee, J., Kim, K. S. & Ramakrishna, S. The Importance of Ubiquitination and Deubiquitination in Cellular Reprogramming. *Stem Cells Int* **2016**, 6705927, doi:10.1155/2016/6705927 (2016).
41 Abashev, T. M., Metzler, M. A., Wright, D. M. & Sandell, L. L. Retinoic acid signaling regulates Krt5 and Krt14 independently of stem cell markers in submandibular salivary gland epithelium. *Dev Dyn* **246**, 135-147, doi:10.1002/dvdy.24476 (2017).
42 Velvarska, H. & Niessing, D. Structural insights into the globular tails of the human type v myosins Myo5a, Myo5b, And Myo5c. *PLoS One* **8**, e82065, doi:10.1371/journal.pone.0082065 (2013).
43 Aranda, J. F. *et al.* MYADM controls endothelial barrier function through ERM-dependent regulation of ICAM-1 expression. *Mol Biol Cell* **24**, 483-494, doi:10.1091/mbc.E11-11-0914 (2013).
44 Aranda, J. F. *et al.* MYADM controls endothelial barrier function through ERM-dependent regulation of ICAM-1 expression. *Mol Biol Cell* **24**, 483-494, doi:10.1091/mbc.E11-11-0914 (2013).
45 Li, P., Hu, J. & Wang, Y. Methods for analyzing histone citrullination in chromatin structure and gene regulation. *Methods Mol Biol* **809**, 473-488, doi:10.1007/978-1-61779-376-9_31 (2012).

46 Zhang, X. *et al.* Peptidylarginine deiminase 1-catalyzed histone citrullination is essential for early embryo development. *Sci Rep* **6**, 38727, doi:10.1038/srep38727 (2016).
47 Jayachandran, P. *et al.* Microtubule-associated protein 1b is required for shaping the neural tube. *Neural Dev* **11**, 1, doi:10.1186/s13064-015-0056-4 (2016).
48 Vervoort, S. J. *et al.* Global transcriptional analysis identifies a novel role for SOX4 in tumor-induced angiogenesis. *Elife* **7**, doi:10.7554/eLife.27706 (2018).
49 Zindel, D. *et al.* Identification of key phosphorylation sites in PTH1R that determine arrestin3 binding and fine-tune receptor signaling. *Biochem J* **473**, 4173-4192, doi:10.1042/BCJ20160740 (2016).
50 Wang, Y. *et al.* Inhibition of PTGS1 promotes osteogenic differentiation of adipose-derived stem cells by suppressing NF-kB signaling. *Stem Cell Res Ther* **10**, 57, doi:10.1186/s13287-019-1167-3 (2019).
51 Balatoni, C. E. *et al.* Epigenetic silencing of Stk39 in B-cell lymphoma inhibits apoptosis from genotoxic stress. *Am J Pathol* **175**, 1653-1661, doi:10.2353/ajpath.2009.090091 (2009).
52 Li, L. *et al.* KLF4-Mediated CDH3 Upregulation Suppresses Human Hepatoma Cell Growth and Migration via GSK-3beta Signaling. *Int J Biol Sci* **15**, 953-961, doi:10.7150/ijbs.30857 (2019).
53 Rivero, O. *et al.* Cadherin-13, a risk gene for ADHD and comorbid disorders, impacts GABAergic function in hippocampus and cognition. *Transl Psychiatry* **5**, e655, doi:10.1038/tp.2015.152 (2015).
54 Schmelzer, C. E. H. *et al.* Lysyl oxidase-like 2 (LOXL2)-mediated cross-linking of tropoelastin. *FASEB J* **33**, 5468-5481, doi:10.1096/fj.201801860RR (2019).
55 Damaghi, M. *et al.* Collagen production and niche engineering: A novel strategy for cancer cells to survive acidosis in DCIS and evolve. *Evol Appl* **13**, 2689-2703, doi:10.1111/eva.13075 (2020).
56 Santana, J. & Marzolo, M. P. The functions of Reelin in membrane trafficking and cytoskeletal dynamics: implications for neuronal migration, polarization and differentiation. *Biochem J* **474**, 3137-3165, doi:10.1042/BCJ20160628 (2017).
57 Bergqvist, F., Morgenstern, R. & Jakobsson, P. J. A review on mPGES-1 inhibitors: From preclinical studies to clinical applications. *Prostaglandins Other Lipid Mediat* **147**, 106383, doi:10.1016/j.prostaglandins.2019.106383 (2020).
58 Lee, J. J. *et al.* Hypoxia activates the cyclooxygenase-2-prostaglandin E synthase axis. *Carcinogenesis* **31**, 427-434, doi:10.1093/carcin/bgp326 (2010).
59 Kuwabara, I. *et al.* Galectin-7 (PIG1) exhibits pro-apoptotic function through JNK activation and mitochondrial cytochrome c release. *J Biol Chem* **277**, 3487-3497, doi:10.1074/jbc.M109360200 (2002).
60 Advedissian, T., Deshayes, F. & Viguier, M. Galectin-7 in Epithelial Homeostasis and Carcinomas. *Int J Mol Sci* **18**, doi:10.3390/ijms18122760 (2017).
61 Britten, J. L., Malik, M., Lewis, T. D. & Catherino, W. H. Ulipristal Acetate Mediates Decreased Proteoglycan Expression Through Regulation of Nuclear Factor of Activated T-Cells (NFAT5). *Reprod Sci* **26**, 184-197, doi:10.1177/1933719118816836 (2019).
62 Clausen, T. M. *et al.* Oncofetal Chondroitin Sulfate Glycosaminoglycans Are Key Players in Integrin Signaling and Tumor Cell Motility. *Mol Cancer Res* **14**, 1288-1299, doi:10.1158/1541-7786.Mcr-16-0103 (2016).

63 Grarup, N. *et al.* Loss-of-function variants in ADCY3 increase risk of obesity and type 2 diabetes. *Nat Genet* **50**, 172-174, doi:10.1038/s41588-017-0022-7 (2018).
64 Guan, Y., Bhandari, A., Zhang, X. & Wang, O. Uridine phosphorylase 1 associates to biological and clinical significance in thyroid carcinoma cell lines. *J Cell Mol Med* **23**, 7438-7448, doi:10.1111/jcmm.14612 (2019).
65 Zerangue, N. & Kavanaugh, M. P. ASCT-1 is a neutral amino acid exchanger with chloride channel activity. *J Biol Chem* **271**, 27991-27994, doi:10.1074/jbc.271.45.27991 (1996).
66 White, M. A. *et al.* Glutamine Transporters Are Targets of Multiple Oncogenic Signaling Pathways in Prostate Cancer. *Mol Cancer Res* **15**, 1017-1028, doi:10.1158/1541-7786.MCR-16-0480 (2017).
67 Eckert, R. L. Sequence of the human 40-kDa keratin reveals an unusual structure with very high sequence identity to the corresponding bovine keratin. *Proc Natl Acad Sci U S A* **85**, 1114-1118, doi:10.1073/pnas.85.4.1114 (1988).
68 Ju, J. H. *et al.* Cytokeratin19 induced by HER2/ERK binds and stabilizes HER2 on cell membranes. *Cell Death Differ* **22**, 665-676, doi:10.1038/cdd.2014.155 (2015).
69 Korpos, E., Deak, F. & Kiss, I. Matrilin-2, an extracellular adaptor protein, is needed for the regeneration of muscle, nerve and other tissues. *Neural Regen Res* **10**, 866-869, doi:10.4103/1673-5374.158332 (2015).
70 Mukhopadhyay, S. *et al.* The ciliary G-protein-coupled receptor Gpr161 negatively regulates the Sonic hedgehog pathway via cAMP signaling. *Cell* **152**, 210-223, doi:10.1016/j.cell.2012.12.026 (2013).
71 Ren, J. *et al.* Overexpression of FNDC1 in Gastric Cancer and its Prognostic Significance. *J Cancer* **9**, 4586-4595, doi:10.7150/jca.27672 (2018).
72 Matsubara, T. *et al.* The Actin-Binding Protein PPP1r18 Regulates Maturation, Actin Organization, and Bone Resorption Activity of Osteoclasts. *Mol Cell Biol* **38**, doi:10.1128/MCB.00425-17 (2018).
73 Jiang, K., Liu, Y., Zhang, J. & Jia, J. An intracellular activation of Smoothened that is independent of Hedgehog stimulation in Drosophila. *J Cell Sci* **131**, doi:10.1242/jcs.211367 (2018).
74 Ehrlich, K. C., Baribault, C. & Ehrlich, M. Epigenetics of Muscle- and Brain-Specific Expression of KLHL Family Genes. *Int J Mol Sci* **21**, doi:10.3390/ijms21218394 (2020).
75 Balenga, N. *et al.* Orphan Adhesion GPCR GPR64/ADGRG2 Is Overexpressed in Parathyroid Tumors and Attenuates Calcium-Sensing Receptor-Mediated Signaling. *J Bone Miner Res* **32**, 654-666, doi:10.1002/jbmr.3023 (2017).
76 Patat, O. *et al.* Truncating Mutations in the Adhesion G Protein-Coupled Receptor G2 Gene ADGRG2 Cause an X-Linked Congenital Bilateral Absence of Vas Deferens. *Am J Hum Genet* **99**, 437-442, doi:10.1016/j.ajhg.2016.06.012 (2016).
77 Sun, Y. *et al.* Optimization of a peptide ligand for the adhesion GPCR ADGRG2 provides a potent tool to explore receptor biology. *J Biol Chem*, doi:10.1074/jbc.RA120.014726 (2020).
78 Groot Kormelink, P. J. & Luyten, W. H. Cloning and sequence of full-length cDNAs encoding the human neuronal nicotinic acetylcholine receptor (nAChR) subunits beta3 and beta4 and expression of seven nAChR subunits in the human neuroblastoma cell line SH-SY5Y and/or IMR-32. *FEBS Lett* **400**, 309-314, doi:10.1016/s0014-5793(96)01383-x (1997).

79 Drayson, L. E. & Triplett, J. W. A Chrnb3-Cre BAC transgenic mouse line for manipulation of gene expression in retinal ganglion cells. *Genesis* **57**, e23305, doi:10.1002/dvg.23305 (2019).
80 Kiso, A. *et al.* Tolloid-Like 1 Negatively Regulates Hepatic Differentiation of Human Induced Pluripotent Stem Cells Through Transforming Growth Factor Beta Signaling. *Hepatol Commun* **4**, 255-267, doi:10.1002/hep4.1466 (2020).
81 Rhodes, K. E. & Fawcett, J. W. Chondroitin sulphate proteoglycans: preventing plasticity or protecting the CNS? *J Anat* **204**, 33-48, doi:10.1111/j.1469-7580.2004.00261.x (2004).
82 Nikolova, Y. S. *et al.* FRAS1-related extracellular matrix 3 (FREM3) single-nucleotide polymorphism effects on gene expression, amygdala reactivity and perceptual processing speed: An accelerated aging pathway of depression risk. *Front Psychol* **6**, 1377, doi:10.3389/fpsyg.2015.01377 (2015).
83 Wang, K. *et al.* Cell-Type-Specific Expression Pattern of Proton-Sensing Receptors and Channels in Pituitary Gland. *Biophys J* **119**, 2335-2348, doi:10.1016/j.bpj.2020.10.013 (2020).
84 Xu, J. *et al.* GPR68 Senses Flow and Is Essential for Vascular Physiology. *Cell* **173**, 762-775 e716, doi:10.1016/j.cell.2018.03.076 (2018).
85 Wiley, S. Z., Sriram, K., Salmeron, C. & Insel, P. A. GPR68: An Emerging Drug Target in Cancer. *Int J Mol Sci* **20**, doi:10.3390/ijms20030559 (2019).
86 Yang, S. *et al.* Cytochrome P-450 epoxygenases protect endothelial cells from apoptosis induced by tumor necrosis factor-alpha via MAPK and PI3K/Akt signaling pathways. *Am J Physiol Heart Circ Physiol* **293**, H142-151, doi:10.1152/ajpheart.00783.2006 (2007).
87 Liu, P. T. *et al.* Toll-like receptor triggering of a vitamin D-mediated human antimicrobial response. *Science* **311**, 1770-1773, doi:10.1126/science.1123933 (2006).
88 Werneburg, S. *et al.* Polysialylation at Early Stages of Oligodendrocyte Differentiation Promotes Myelin Repair. *J Neurosci* **37**, 8131-8141, doi:10.1523/JNEUROSCI.1147-17.2017 (2017).
89 Dennis, J., Waller, C., Timpl, R. & Schirrmacher, V. Surface sialic acid reduces attachment of metastatic tumour cells to collagen type IV and fibronectin. *Nature* **300**, 274-276, doi:10.1038/300274a0 (1982).
90 Mylonis, I., Simos, G. & Paraskeva, E. Hypoxia-Inducible Factors and the Regulation of Lipid Metabolism. *Cells* **8**, doi:10.3390/cells8030214 (2019).
91 Li, S. H. *et al.* An actin-binding protein ESPN is an independent prognosticator and regulates cell growth for esophageal squamous cell carcinoma. *Cancer Cell Int* **18**, 219, doi:10.1186/s12935-018-0713-x (2018).
92 Tan, Y. *et al.* The homeoprotein Dlx5 drives murine T-cell lymphomagenesis by directly transactivating Notch and upregulating Akt signaling. *Oncotarget* **8**, 14941-14956, doi:10.18632/oncotarget.14784 (2017).
93 Meyerhardt, J. A. *et al.* Netrin-1: interaction with deleted in colorectal cancer (DCC) and alterations in brain tumors and neuroblastomas. *Cell Growth Differ* **10**, 35-42 (1999).
94 Grandin, M. *et al.* Inhibition of DNA methylation promotes breast tumor sensitivity to netrin-1 interference. *EMBO Mol Med* **8**, 863-877, doi:10.15252/emmm.201505945 (2016).

95 Porter, L. F. *et al.* A role for repressive complexes and H3K9 di-methylation in PRDM5-associated brittle cornea syndrome. *Hum Mol Genet* **24**, 6565-6579, doi:10.1093/hmg/ddv345 (2015).
96 Yao, L. L. *et al.* Astrocytic neogenin/netrin-1 pathway promotes blood vessel homeostasis and function in mouse cortex. *J Clin Invest* **130**, 6490-6509, doi:10.1172/jci132372 (2020).
97 Wilson, L. H. *et al.* Liver Glycogen Phosphorylase Deficiency Leads to Profibrogenic Phenotype in a Murine Model of Glycogen Storage Disease Type VI. *Hepatol Commun* **3**, 1544-1555, doi:10.1002/hep4.1426 (2019).
98 Luo, X. *et al.* Novel PYGL mutations in Chinese children leading to glycogen storage disease type VI: two case reports. *BMC Med Genet* **21**, 74, doi:10.1186/s12881-020-01010-4 (2020).
99 Man, M. Q. *et al.* Basis for enhanced barrier function of pigmented skin. *J Invest Dermatol* **134**, 2399-2407, doi:10.1038/jid.2014.187 (2014).
100 Matesic, L. E. *et al.* Mutations in Mlph, encoding a member of the Rab effector family, cause the melanosome transport defects observed in leaden mice. *Proc Natl Acad Sci U S A* **98**, 10238-10243, doi:10.1073/pnas.181336698 (2001).
101 Robinson, C. L. *et al.* The adaptor protein melanophilin regulates dynamic myosin-Va:cargo interaction and dendrite development in melanocytes. *Mol Biol Cell* **30**, 742-752, doi:10.1091/mbc.E18-04-0237 (2019).
102 Mishra, N. K. *et al.* FXYD proteins stabilize Na,K-ATPase: amplification of specific phosphatidylserine-protein interactions. *J Biol Chem* **286**, 9699-9712, doi:10.1074/jbc.M110.184234 (2011).
103 Lubarski, I., Karlish, S. J. & Garty, H. Structural and functional interactions between FXYD5 and the Na+-K+-ATPase. *Am J Physiol Renal Physiol* **293**, F1818-1826, doi:10.1152/ajprenal.00367.2007 (2007).
104 Degrandi, D. *et al.* Extensive characterization of IFN-induced GTPases mGBP1 to mGBP10 involved in host defense. *J Immunol* **179**, 7729-7740, doi:10.4049/jimmunol.179.11.7729 (2007).
105 Degrandi, D. *et al.* Murine guanylate binding protein 2 (mGBP2) controls Toxoplasma gondii replication. *Proc Natl Acad Sci U S A* **110**, 294-299, doi:10.1073/pnas.1205635110 (2013).
106 Artegiani, B. *et al.* Tox: a multifunctional transcription factor and novel regulator of mammalian corticogenesis. *EMBO J* **34**, 896-910, doi:10.15252/embj.201490061 (2015).
107 Vorkapic, E., Kunath, A. & Wagsater, D. Effects of osteoprotegerin/TNFRSF11B in two models of abdominal aortic aneurysms. *Mol Med Rep* **18**, 41-48, doi:10.3892/mmr.2018.8936 (2018).
108 Cawley, K. M. *et al.* Local Production of Osteoprotegerin by Osteoblasts Suppresses Bone Resorption. *Cell Rep* **32**, 108052, doi:10.1016/j.celrep.2020.108052 (2020).
109 White, S. N. *et al.* Genome-wide association identifies multiple genomic regions associated with susceptibility to and control of ovine lentivirus. *PLoS One* **7**, e47829, doi:10.1371/journal.pone.0047829 (2012).
110 Kong, W., Longaker, M. T. & Lorenz, H. P. Molecular cloning and expression of keratinocyte proline-rich protein, a novel squamous epithelial marker isolated during skin development. *J Biol Chem* **278**, 22781-22786, doi:10.1074/jbc.M210488200 (2003).

111 Nissila, E. *et al.* C4B gene influences intestinal microbiota through complement activation in patients with paediatric-onset inflammatory bowel disease. *Clin Exp Immunol* **190**, 394-405, doi:10.1111/cei.13040 (2017).
112 Uyama, T., Kitagawa, H., Tamura Ji, J. & Sugahara, K. Molecular cloning and expression of human chondroitin N-acetylgalactosaminyltransferase: the key enzyme for chain initiation and elongation of chondroitin/dermatan sulfate on the protein linkage region tetrasaccharide shared by heparin/heparan sulfate. *J Biol Chem* **277**, 8841-8846, doi:10.1074/jbc.M111434200 (2002).
113 Mizumoto, S. *et al.* CSGALNACT1-congenital disorder of glycosylation: A mild skeletal dysplasia with advanced bone age. *Hum Mutat* **41**, 655-667, doi:10.1002/humu.23952 (2020).
114 Onishi, K. & Zou, Y. Sonic Hedgehog switches on Wnt/planar cell polarity signaling in commissural axon growth cones by reducing levels of Shisa2. *Elife* **6**, doi:10.7554/eLife.25269 (2017).
115 Desai, B. S., Chadha, A. & Cook, B. The stum gene is essential for mechanical sensing in proprioceptive neurons. *Science* **343**, 1256-1259, doi:10.1126/science.1247761 (2014).
116 Chen, C., Jiang, L., Zhang, Y. & Zheng, W. FOXA1-induced LINC01207 facilitates head and neck squamous cell carcinoma via up-regulation of TNRC6B. *Biomed Pharmacother* **128**, 110220, doi:10.1016/j.biopha.2020.110220 (2020).
117 Plun-Favreau, H. *et al.* The ciliary neurotrophic factor receptor alpha component induces the secretion of and is required for functional responses to cardiotrophin-like cytokine. *EMBO J* **20**, 1692-1703, doi:10.1093/emboj/20.7.1692 (2001).
118 Cox-Limpens, K. E., Vles, J. S., D, L. A. v. d. H., Zimmermann, L. J. & Gavilanes, A. W. Fetal asphyctic preconditioning alters the transcriptional response to perinatal asphyxia. *BMC Neurosci* **15**, 67, doi:10.1186/1471-2202-15-67 (2014).
119 Katsu, Y. & Iguchi, T. Tissue-specific expression of Clec2g in mice. *Eur J Cell Biol* **85**, 345-354, doi:10.1016/j.ejcb.2005.12.004 (2006).
120 Ekici, A. B. *et al.* Disturbed Wnt Signalling due to a Mutation in CCDC88C Causes an Autosomal Recessive Non-Syndromic Hydrocephalus with Medial Diverticulum. *Mol Syndromol* **1**, 99-112, doi:10.1159/000319859 (2010).
121 Ear, J. *et al.* Tyrosine-Based Signals Regulate the Assembly of Daple·PARD3 Complex at Cell-Cell Junctions. *iScience* **23**, 100859, doi:10.1016/j.isci.2020.100859 (2020).
122 Shastry, S. *et al.* Deletion in the A4GALT Gene Associated with Rare "P null" Phenotype: The First Report from India. *Transfus Med Hemother* **47**, 186-189, doi:10.1159/000501916 (2020).
123 Gao, S. *et al.* The interaction between flagellin and the glycosphingolipid Gb3 on host cells contributes to Bacillus cereus acute infection. *Virulence* **11**, 769-780, doi:10.1080/21505594.2020.1773077 (2020).
124 Giessner, C. *et al.* Vnn1 pantetheinase limits the Warburg effect and sarcoma growth by rescuing mitochondrial activity. *Life Sci Alliance* **1**, e201800073, doi:10.26508/lsa.201800073 (2018).
125 Ferreira, D. W., Naquet, P. & Manautou, J. E. Influence of Vanin-1 and Catalytic Products in Liver During Normal and Oxidative Stress Conditions. *Curr Med Chem* **22**, 2407-2416, doi:10.2174/0929867322220150722124307 (2015).

126 Punetha, J. *et al.* Biallelic CACNA2D2 variants in epileptic encephalopathy and cerebellar atrophy. *Ann Clin Transl Neurol* **6**, 1395-1406, doi:10.1002/acn3.50824 (2019).
127 Lodge, E. J. *et al.* Requirement of FAT and DCHS protocadherins during hypothalamic-pituitary development. *JCI Insight* **5**, doi:10.1172/jci.insight.134310 (2020).
128 Karrys, A. *et al.* Bioactive Dietary VDR Ligands Regulate Genes Encoding Biomarkers of Skin Repair That Are Associated with Risk for Psoriasis. *Nutrients* **10**, doi:10.3390/nu10020174 (2018).
129 Pai, Y. J. *et al.* Glycine decarboxylase deficiency causes neural tube defects and features of non-ketotic hyperglycinemia in mice. *Nat Commun* **6**, 6388, doi:10.1038/ncomms7388 (2015).
130 Diaz-Horta, O. *et al.* Ripor2 is involved in auditory hair cell stereociliary bundle structure and orientation. *J Mol Med (Berl)* **96**, 1227-1238, doi:10.1007/s00109-018-1694-x (2018).
131 Huang, X. *et al.* NOV/CCN3 induces cartilage protection by inhibiting PI3K/AKT/mTOR pathway. *J Cell Mol Med* **23**, 7525-7534, doi:10.1111/jcmm.14621 (2019).
132 Kuwahara, M. *et al.* CCN3 (NOV) Drives Degradative Changes in Aging Articular Cartilage. *Int J Mol Sci* **21**, doi:10.3390/ijms21207556 (2020).
133 Sreedharan, S. *et al.* The G protein coupled receptor Gpr153 shares common evolutionary origin with Gpr162 and is highly expressed in central regions including the thalamus, cerebellum and the arcuate nucleus. *FEBS J* **278**, 4881-4894, doi:10.1111/j.1742-4658.2011.08388.x (2011).
134 Li, P. *et al.* Novel Therapy for Glioblastoma Multiforme by Restoring LRRC4 in Tumor Cells: LRRC4 Inhibits Tumor-Infitrating Regulatory T Cells by Cytokine and Programmed Cell Death 1-Containing Exosomes. *Front Immunol* **8**, 1748, doi:10.3389/fimmu.2017.01748 (2017).
135 Zhao, C. *et al.* LRRC4 Suppresses E-Cadherin-Dependent Collective Cell Invasion and Metastasis in Epithelial Ovarian Cancer. *Front Oncol* **10**, 144, doi:10.3389/fonc.2020.00144 (2020).
136 Seda, O. *et al.* ZBTB16 and metabolic syndrome: a network perspective. *Physiol Res* **66**, S357-S365, doi:10.33549/physiolres.933730 (2017).
137 He, H. *et al.* Study on the mechanism behind lncRNA MEG3 affecting clear cell renal cell carcinoma by regulating miR-7/RASL11B signaling. *J Cell Physiol* **233**, 9503-9515, doi:10.1002/jcp.26849 (2018).
138 Ligthart-Melis, G. C. & Deutz, N. E. Is glutamine still an important precursor of citrulline? *Am J Physiol Endocrinol Metab* **301**, E264-266, doi:10.1152/ajpendo.00223.2011 (2011).
139 Hannemann, J., Zummack, J., Hillig, J. & Boger, R. Metabolism of asymmetric dimethylarginine in hypoxia: from bench to bedside. *Pulm Circ* **10**, 2045894020918846, doi:10.1177/2045894020918846 (2020).
140 Hannemann, J. *et al.* Upregulation of DDAH2 Limits Pulmonary Hypertension and Right Ventricular Hypertrophy During Chronic Hypoxia in Ddah1 Knockout Mice. *Front Physiol* **11**, 597559, doi:10.3389/fphys.2020.597559 (2020).

Suppl. Table 2. Oligonucleotide primers and expected amplicon size used for qReal Time RT-PCR analysis.

Genes	Primers - 5’→3’	Amplicon size
pygl	Forward: GCCTGGAACACAATGGTACT<br>Reverse: GGACTCGTTGGATAGGGAAATC	135
espn	Forward: CAAAGTGAGAGTCCTGAGACAC<br>Reverse: CGCCTTTATCTCAGCCAGAA	97
tubb4a	Forward: ATGAGGCCACAGGTGGAAAC<br>Reverse: CTCGGTGTAGTGACCCTTGG	169
frem3	Forward: GGCCGACTGTGAGGCTTTTAT<br>Reverse: GCATGGGCACATAGTCCCTG	100
st8sia4	Forward: GGTGGACCATCTGCACTATAAG<br>Reverse: GATGAGTTGCGTCTCTTGGT	92
tll1	Forward: CGTGCAAAGCTGCTGTGTTT<br>Reverse: GCATGCCATGGTCTCCAAAG	146
glyat	Forward: TGAGAGGAGCCAGAGATTCA<br>Reverse: CTCTCCAGTTTGGTCCATTAGAG	112
mmrn1	Forward: GCCCTTACAGCGATTCCTAAA<br>Reverse: CACCTGGGAAGTGGTTTCAT	90
tcf4	Forward: GTTTGAGCTATCCATCCCACTC<br>Reverse: GCAGGAAGAGGTGCTGTAAT	104
slc4a11	Forward: CTGTGCTGTATGGGCTCTTT<br>Reverse: GGTATGATGTCTGCTCCTTGAG	102
col8a2	Forward: CACCTACACGTACGACGAATAC<br>Reverse: TCGGTGGAGTAGAGACCATT	135
lamc1	Forward: GTTACCGACTGGTGAAGGATAAG<br>Reverse: TGTCCTCAAAGGCTTGATCTG	120
col14a1	Forward: GAAGTGGTAGGAACGGTTATGG<br>Reverse: GACGCCTTCTCCAACAGTATAG	120
gapdh	Forward: AACAGCAACTCCCACTCTTC<br>Reverse: CCTGTTGCTGTAGCCGTATT	110

Suppl. Table 3. Altered gene expression in *slc4a11*$^{-/-}$ mice and human FECD corneas. The 1556 genes with altered expression level in human FECD corneas[1] were compared to altered genes found in the present manuscript. Genes with significantly altered expression in both studies are listed in the table.

Mouse gene	Human Gene	Gene function
ccdc88c	*CCDC80*	CCDC88C encodes DAPLE, a regulator of epithelial cell polarity[122,123]
ripor2 (also called fam65b	*FAM65B*	RHO family interacting cell polarization regulator 2 (RIPOR2, previously known as FAM65B) is an essential protein required in human hearing, that acts as an inhibitor of the small G protein RhoA[132].<br>Regulates abundance and post translational modifications of proteins expressed in the kinocilium.
fhl3	*FHL2*	Four-and-a-half LIM domains 3 (FHL3) inhibits regulates epithelial-mesenchymal transition (EMT)[8].<br>Regulator of myoblast differentiation- acts as a transcriptional activator or repressor[9].
krt4	*KRT8*	Keratin 4 (KRT4) forms intermediate filaments, a structural component of the cytoskeleton in all epithelial cells[32].
map1b	*MAP7D1*	Microtubule associated protein 1b (MAP1B) is important in microtubule assembly[49].
myo5a	*MYO18A*	Myosin gene superfamily member 5A (MYO5A) is one of the three myosin V heavy-chain genes, part of an actin-based motor protein.<br>Myo5a can dissociate from the ribosome complex to release ribosomes from the actin cytoskeleton[44].
nol4	*NOL3 and NOLC1*	Nucleolar protein 4 (NOL4) is involved in determining cell specificity. Abundant in malignant cells[19].
nudt11	*NUDT4*	Nucleoside diphosphate attached moiety 'X'-type (NUDT) is a phosphohydrolase that hydrolyses 5-phosphoribosyl 1-pyrophosphate (PRPP) to generate glycolytic activator, ribose 1,5 bisphosphate[10].
ppplrl8	*PPPLR7*	Protein phosphatase 1 regulatory subunit 18 (PPP1r18) overexpression negatively regulates actin ring formation.<br>Suppresses actin elongation by directly binding to actin[74].
stk39	*STK16*	STE20 (sterile 20-like)-related proline-alanine-rich kinase (SPAK) is encoded by *STK39*. STE20 kinases are important in osmotic stress signaling. SPAK is ubiquitously expressed and modulates ion and fluid homeostasis through phosphorylation of chloride transporters and p38 MAPK pathway activation[53].
tnfrsf11b	*TNFRSF25*	Tumor necrosis factor receptor superfamily member 11B (*TNFRSF11B*) encodes osteoprotegerin, a decoy receptor for receptor activator of NF-κB ligand (RANKL)[109,110].
tshz3 (also called znf537)	*ZNF207, ZNF219, ZNF638*	Teashirt zinc finger homeobox family member 3 (TSHZ3; also known as ZNF537) encodes a zinc finger transcription factor upregulated upon oxidative damage[15,16].<br>TSHZ3 overexpression in myoblasts inhibited myogenic differentiation and decreased Myog, a transcription factor that induces myogenesis, expression.

References

1 Wieben, E. D. *et al.* Gene Expression and Missplicing in the Corneal Endothelium of Patients With a TCF4 Trinucleotide Repeat Expansion Without Fuchs' Endothelial Corneal Dystrophy. *Invest Ophthalmol Vis Sci* **60**, 3636-3643, doi:10.1167/iovs.19-27689 (2019).

2 Frausto, R. F., Le, D. J. & Aldave, A. J. Transcriptomic Analysis of Cultured Corneal Endothelial Cells as a Validation for Their Use in Cell Replacement Therapy. *Cell Transplant* **25**, 1159-1176, doi:10.3727/096368915x688948 (2016).

3 Bohnsack, J. P., Patel, V. K. & Morrow, A. L. Ethanol Exposure Regulates Gabra1 Expression via Histone Deacetylation at the Promoter in Cultured Cortical Neurons. *J Pharmacol Exp Ther* **363**, 1-11, doi:10.1124/jpet.117.242446 (2017).

4 Zhang, T. *et al.* Meta-analysis of GABRB2 polymorphisms and the risk of schizophrenia combined with GWAS data of the Han Chinese population and psychiatric genomics consortium. *PLoS One* **13**, e0198690, doi:10.1371/journal.pone.0198690 (2018).

5 Berger, T. C. *et al.* Neuronal and glial DNA methylation and gene expression changes in early epileptogenesis. *PLoS One* **14**, e0226575, doi:10.1371/journal.pone.0226575 (2019).

6 Roman, D., Zhong, H., Yaklichkin, S., Chen, R. & Mardon, G. Conditional loss of Kcnj13 in the retinal pigment epithelium causes photoreceptor degeneration. *Exp Eye Res* **176**, 219-226, doi:10.1016/j.exer.2018.07.014 (2018).

7 Parker, D. N. *et al.* The functions of the A1A2A3 domains in von Willebrand factor include multimerin 1 binding. *Thromb Haemost* **116**, 87-95, doi:10.1160/TH15-09-0700 (2016).

8 Li, P. *et al.* FHL3 promotes pancreatic cancer invasion and metastasis through preventing the ubiquitination degradation of EMT associated transcription factors. *Aging (Albany NY)* **12**, 53-69, doi:10.18632/aging.102564 (2020).

9 Zhang, Y. *et al.* FHL3 differentially regulates the expression of MyHC isoforms through interactions with MyoD and pCREB. *Cell Signal* **28**, 60-73, doi:10.1016/j.cellsig.2015.10.008 (2016).

10 Hua, L. V., Hidaka, K., Pesesse, X., Barnes, L. D. & Shears, S. B. Paralogous murine Nudt10 and Nudt11 genes have differential expression patterns but encode identical proteins that are physiologically competent diphosphoinositol polyphosphate phosphohydrolases. *Biochem J* **373**, 81-89, doi:10.1042/BJ20030142 (2003).

11 Valkenburg, K. C., Graveel, C. R., Zylstra-Diegel, C. R., Zhong, Z. & Williams, B. O. Wnt/beta-catenin Signaling in Normal and Cancer Stem Cells. *Cancers (Basel)* **3**, 2050-2079, doi:10.3390/cancers3022050 (2011).

12 Collette, N. M. *et al.* Sost and its paralog Sostdc1 coordinate digit number in a Gli3-dependent manner. *Dev Biol* **383**, 90-105, doi:10.1016/j.ydbio.2013.08.015 (2013).

13 Kim, Y. S., Nakanishi, G., Lewandoski, M. & Jetten, A. M. GLIS3, a novel member of the GLIS subfamily of Kruppel-like zinc finger proteins with repressor and activation functions. *Nucleic Acids Res* **31**, 5513-5525, doi:10.1093/nar/gkg776 (2003).

14 Jetten, A. M. GLIS1-3 transcription factors: critical roles in the regulation of multiple physiological processes and diseases. *Cell Mol Life Sci* **75**, 3473-3494, doi:10.1007/s00018-018-2841-9 (2018).

15 Faralli, H. *et al.* Teashirt-3, a novel regulator of muscle differentiation, associates with BRG1-associated factor 57 (BAF57) to inhibit myogenin gene expression. *J Biol Chem* **286**, 23498-23510, doi:10.1074/jbc.M110.206003 (2011).

16 Meng, X., Zhu, Y., Tao, L., Zhao, S. & Qiu, S. MicroRNA-125b-1-3p mediates intervertebral disc degeneration in rats by targeting teashirt zinc finger homeobox 3. *Exp Ther Med* **15**, 2627-2633, doi:10.3892/etm.2018.5715 (2018).

17 Kordass, T., Osen, W. & Eichmuller, S. B. Controlling the Immune Suppressor: Transcription Factors and MicroRNAs Regulating CD73/NT5E. *Front Immunol* **9**, 813, doi:10.3389/fimmu.2018.00813 (2018).

18 Viard, P. *et al.* PI3K promotes voltage-dependent calcium channel trafficking to the plasma membrane. *Nat Neurosci* **7**, 939-946, doi:10.1038/nn1300 (2004).

19 Zasada, M. *et al.* Short- and long-term impact of hyperoxia on the blood and retinal cells' transcriptome in a mouse model of oxygen-induced retinopathy. *Pediatr Res* **87**, 485-493, doi:10.1038/s41390-019-0598-y (2020).

20 Bitoun, E. *et al.* Netherton syndrome: disease expression and spectrum of SPINK5 mutations in 21 families. *J Invest Dermatol* **118**, 352-361, doi:10.1046/j.1523-1747.2002.01603.x (2002).

21 Chavanas, S. *et al.* Mutations in SPINK5, encoding a serine protease inhibitor, cause Netherton syndrome. *Nat Genet* **25**, 141-142, doi:10.1038/75977 (2000).

22 Veldurthy, V. *et al.* Vitamin D, calcium homeostasis and aging. *Bone Res* **4**, 16041, doi:10.1038/boneres.2016.41 (2016).

23 Cushion, T. D. *et al.* De novo mutations in the beta-tubulin gene TUBB2A cause simplified gyral patterning and infantile-onset epilepsy. *Am J Hum Genet* **94**, 634-641, doi:10.1016/j.ajhg.2014.03.009 (2014).

24 Xiao, J., Vemula, S. R. & LeDoux, M. S. Recent advances in the genetics of dystonia. *Curr Neurol Neurosci Rep* **14**, 462, doi:10.1007/s11910-014-0462-8 (2014).

25 Guo, F. *et al.* Correlation Between TNFAIP2 Gene Polymorphism and Prediction/Prognosis for Gastric Cancer and Its Effect on TNFAIP2 Protein Expression. *Front Oncol* **10**, 1127, doi:10.3389/fonc.2020.01127 (2020).

26 Liang, X., Kiru, S., Gomez, G. A. & Yap, A. S. Regulated recruitment of SRGAP1 modulates RhoA signaling for contractility during epithelial junction maturation. *Cytoskeleton (Hoboken)* **75**, 61-69, doi:10.1002/cm.21420 (2018).

27 Yamazaki, D., Itoh, T., Miki, H. & Takenawa, T. srGAP1 regulates lamellipodial dynamics and cell migratory behavior by modulating Rac1 activity. *Mol Biol Cell* **24**, 3393-3405, doi:10.1091/mbc.E13-04-0178 (2013).

28 Radichev, I. A. *et al.* Nardilysin-dependent proteolysis of cell-associated VTCN1 (B7-H4) marks type 1 diabetes development. *Diabetes* **63**, 3470-3482, doi:10.2337/db14-0213 (2014).

29 Sheikh, A. *et al.* Unfavorable neuroblastoma prognostic factor NLRR2 inhibits cell differentiation by transcriptional induction through JNK pathway. *Cancer Sci* **107**, 1223-1232, doi:10.1111/cas.13003 (2016).

30 Dunn, H. A., Patil, D. N., Cao, Y., Orlandi, C. & Martemyanov, K. A. Synaptic adhesion protein ELFN1 is a selective allosteric modulator of group III metabotropic glutamate receptors in trans. *Proc Natl Acad Sci U S A* **115**, 5022-5027, doi:10.1073/pnas.1722498115 (2018).

31 Park, D. H. *et al.* N-linked glycosylation of the mGlu7 receptor regulates the forward trafficking and transsynaptic interaction with Elfn1. *Faseb j* **34**, 14977-14996, doi:10.1096/fj.202001544R (2020).
32 Kurklu, E. *et al.* Clinical features and molecular genetic analysis in a Turkish family with oral white sponge nevus. *Med Oral Patol Oral Cir Bucal* **23**, e144-e150, doi:10.4317/medoral.21437 (2018).
33 Villari, G. *et al.* A direct interaction between fascin and microtubules contributes to adhesion dynamics and cell migration. *J Cell Sci* **128**, 4601-4614, doi:10.1242/jcs.175760 (2015).
34 Johansson, M., Giger, F. A., Fielding, T. & Houart, C. Dkk1 Controls Cell-Cell Interaction through Regulation of Non-nuclear beta-Catenin Pools. *Dev Cell* **51**, 775-786 e773, doi:10.1016/j.devcel.2019.10.026 (2019).
35 Vegh, A. *et al.* Comprehensive Analysis of DWARF14-LIKE2 (DLK2) Reveals Its Functional Divergence from Strigolactone-Related Paralogs. *Front Plant Sci* **8**, 1641, doi:10.3389/fpls.2017.01641 (2017).
36 Hemmavanh, C., Koch, M., Birk, D. E. & Espana, E. M. Abnormal corneal endothelial maturation in collagen XII and XIV null mice. *Invest Ophthalmol Vis Sci* **54**, 3297-3308, doi:10.1167/iovs.12-11456 (2013).
37 Guo, Y. F. *et al.* Suggestion of GLYAT gene underlying variation of bone size and body lean mass as revealed by a bivariate genome-wide association study. *Hum Genet* **132**, 189-199, doi:10.1007/s00439-012-1236-5 (2013).
38 Matsuyama, S. *et al.* Interaction between cardiac myosin-binding protein C and formin Fhod3. *Proc Natl Acad Sci U S A* **115**, E4386-E4395, doi:10.1073/pnas.1716498115 (2018).
39 Wang, L. *et al.* AREG mediates the epithelialmesenchymal transition in pancreatic cancer cells via the EGFR/ERK/NFkappaB signalling pathway. *Oncol Rep* **43**, 1558-1568, doi:10.3892/or.2020.7523 (2020).
40 Stoll, S. W. *et al.* Membrane-Tethered Intracellular Domain of Amphiregulin Promotes Keratinocyte Proliferation. *J Invest Dermatol* **136**, 444-452, doi:10.1016/j.jid.2015.10.061 (2016).
41 Lin, Z. *et al.* Stabilizing mutations of KLHL24 ubiquitin ligase cause loss of keratin 14 and human skin fragility. *Nat Genet* **48**, 1508-1516, doi:10.1038/ng.3701 (2016).
42 Suresh, B., Lee, J., Kim, K. S. & Ramakrishna, S. The Importance of Ubiquitination and Deubiquitination in Cellular Reprogramming. *Stem Cells Int* **2016**, 6705927, doi:10.1155/2016/6705927 (2016).
43 Abashev, T. M., Metzler, M. A., Wright, D. M. & Sandell, L. L. Retinoic acid signaling regulates Krt5 and Krt14 independently of stem cell markers in submandibular salivary gland epithelium. *Dev Dyn* **246**, 135-147, doi:10.1002/dvdy.24476 (2017).
44 Velvarska, H. & Niessing, D. Structural insights into the globular tails of the human type v myosins Myo5a, Myo5b, And Myo5c. *PLoS One* **8**, e82065, doi:10.1371/journal.pone.0082065 (2013).
45 Aranda, J. F. *et al.* MYADM controls endothelial barrier function through ERM-dependent regulation of ICAM-1 expression. *Mol Biol Cell* **24**, 483-494, doi:10.1091/mbc.E11-11-0914 (2013).

46 Aranda, J. F. *et al.* MYADM controls endothelial barrier function through ERM-dependent regulation of ICAM-1 expression. *Mol Biol Cell* **24**, 483-494, doi:10.1091/mbc.E11-11-0914 (2013).

47 Li, P., Hu, J. & Wang, Y. Methods for analyzing histone citrullination in chromatin structure and gene regulation. *Methods Mol Biol* **809**, 473-488, doi:10.1007/978-1-61779-376-9_31 (2012).

48 Zhang, X. *et al.* Peptidylarginine deiminase 1-catalyzed histone citrullination is essential for early embryo development. *Sci Rep* **6**, 38727, doi:10.1038/srep38727 (2016).

49 Jayachandran, P. *et al.* Microtubule-associated protein 1b is required for shaping the neural tube. *Neural Dev* **11**, 1, doi:10.1186/s13064-015-0056-4 (2016).

50 Vervoort, S. J. *et al.* Global transcriptional analysis identifies a novel role for SOX4 in tumor-induced angiogenesis. *Elife* **7**, doi:10.7554/eLife.27706 (2018).

51 Zindel, D. *et al.* Identification of key phosphorylation sites in PTH1R that determine arrestin3 binding and fine-tune receptor signaling. *Biochem J* **473**, 4173-4192, doi:10.1042/BCJ20160740 (2016).

52 Wang, Y. *et al.* Inhibition of PTGS1 promotes osteogenic differentiation of adipose-derived stem cells by suppressing NF-kB signaling. *Stem Cell Res Ther* **10**, 57, doi:10.1186/s13287-019-1167-3 (2019).

53 Balatoni, C. E. *et al.* Epigenetic silencing of Stk39 in B-cell lymphoma inhibits apoptosis from genotoxic stress. *Am J Pathol* **175**, 1653-1661, doi:10.2353/ajpath.2009.090091 (2009).

54 Li, L. *et al.* KLF4-Mediated CDH3 Upregulation Suppresses Human Hepatoma Cell Growth and Migration via GSK-3beta Signaling. *Int J Biol Sci* **15**, 953-961, doi:10.7150/ijbs.30857 (2019).

55 Rivero, O. *et al.* Cadherin-13, a risk gene for ADHD and comorbid disorders, impacts GABAergic function in hippocampus and cognition. *Transl Psychiatry* **5**, e655, doi:10.1038/tp.2015.152 (2015).

56 Schmelzer, C. E. H. *et al.* Lysyl oxidase-like 2 (LOXL2)-mediated cross-linking of tropoelastin. *FASEB J* **33**, 5468-5481, doi:10.1096/fj.201801860RR (2019).

57 Damaghi, M. *et al.* Collagen production and niche engineering: A novel strategy for cancer cells to survive acidosis in DCIS and evolve. *Evol Appl* **13**, 2689-2703, doi:10.1111/eva.13075 (2020).

58 Santana, J. & Marzolo, M. P. The functions of Reelin in membrane trafficking and cytoskeletal dynamics: implications for neuronal migration, polarization and differentiation. *Biochem J* **474**, 3137-3165, doi:10.1042/BCJ20160628 (2017).

59 Bergqvist, F., Morgenstern, R. & Jakobsson, P. J. A review on mPGES-1 inhibitors: From preclinical studies to clinical applications. *Prostaglandins Other Lipid Mediat* **147**, 106383, doi:10.1016/j.prostaglandins.2019.106383 (2020).

60 Lee, J. J. *et al.* Hypoxia activates the cyclooxygenase-2-prostaglandin E synthase axis. *Carcinogenesis* **31**, 427-434, doi:10.1093/carcin/bgp326 (2010).

61 Kuwabara, I. *et al.* Galectin-7 (PIG1) exhibits pro-apoptotic function through JNK activation and mitochondrial cytochrome c release. *J Biol Chem* **277**, 3487-3497, doi:10.1074/jbc.M109360200 (2002).

62 Advedissian, T., Deshayes, F. & Viguier, M. Galectin-7 in Epithelial Homeostasis and Carcinomas. *Int J Mol Sci* **18**, doi:10.3390/ijms18122760 (2017).

63 Britten, J. L., Malik, M., Lewis, T. D. & Catherino, W. H. Ulipristal Acetate Mediates Decreased Proteoglycan Expression Through Regulation of Nuclear Factor of Activated T-Cells (NFAT5). *Reprod Sci* **26**, 184-197, doi:10.1177/1933719118816836 (2019).
64 Clausen, T. M. *et al.* Oncofetal Chondroitin Sulfate Glycosaminoglycans Are Key Players in Integrin Signaling and Tumor Cell Motility. *Mol Cancer Res* **14**, 1288-1299, doi:10.1158/1541-7786.Mcr-16-0103 (2016).
65 Grarup, N. *et al.* Loss-of-function variants in ADCY3 increase risk of obesity and type 2 diabetes. *Nat Genet* **50**, 172-174, doi:10.1038/s41588-017-0022-7 (2018).
66 Guan, Y., Bhandari, A., Zhang, X. & Wang, O. Uridine phosphorylase 1 associates to biological and clinical significance in thyroid carcinoma cell lines. *J Cell Mol Med* **23**, 7438-7448, doi:10.1111/jcmm.14612 (2019).
67 Zerangue, N. & Kavanaugh, M. P. ASCT-1 is a neutral amino acid exchanger with chloride channel activity. *J Biol Chem* **271**, 27991-27994, doi:10.1074/jbc.271.45.27991 (1996).
68 White, M. A. *et al.* Glutamine Transporters Are Targets of Multiple Oncogenic Signaling Pathways in Prostate Cancer. *Mol Cancer Res* **15**, 1017-1028, doi:10.1158/1541-7786.MCR-16-0480 (2017).
69 Eckert, R. L. Sequence of the human 40-kDa keratin reveals an unusual structure with very high sequence identity to the corresponding bovine keratin. *Proc Natl Acad Sci U S A* **85**, 1114-1118, doi:10.1073/pnas.85.4.1114 (1988).
70 Ju, J. H. *et al.* Cytokeratin19 induced by HER2/ERK binds and stabilizes HER2 on cell membranes. *Cell Death Differ* **22**, 665-676, doi:10.1038/cdd.2014.155 (2015).
71 Korpos, E., Deak, F. & Kiss, I. Matrilin-2, an extracellular adaptor protein, is needed for the regeneration of muscle, nerve and other tissues. *Neural Regen Res* **10**, 866-869, doi:10.4103/1673-5374.158332 (2015).
72 Mukhopadhyay, S. *et al.* The ciliary G-protein-coupled receptor Gpr161 negatively regulates the Sonic hedgehog pathway via cAMP signaling. *Cell* **152**, 210-223, doi:10.1016/j.cell.2012.12.026 (2013).
73 Ren, J. *et al.* Overexpression of FNDC1 in Gastric Cancer and its Prognostic Significance. *J Cancer* **9**, 4586-4595, doi:10.7150/jca.27672 (2018).
74 Matsubara, T. *et al.* The Actin-Binding Protein PPP1r18 Regulates Maturation, Actin Organization, and Bone Resorption Activity of Osteoclasts. *Mol Cell Biol* **38**, doi:10.1128/MCB.00425-17 (2018).
75 Jiang, K., Liu, Y., Zhang, J. & Jia, J. An intracellular activation of Smoothened that is independent of Hedgehog stimulation in Drosophila. *J Cell Sci* **131**, doi:10.1242/jcs.211367 (2018).
76 Ehrlich, K. C., Baribault, C. & Ehrlich, M. Epigenetics of Muscle- and Brain-Specific Expression of KLHL Family Genes. *Int J Mol Sci* **21**, doi:10.3390/ijms21218394 (2020).
77 Balenga, N. *et al.* Orphan Adhesion GPCR GPR64/ADGRG2 Is Overexpressed in Parathyroid Tumors and Attenuates Calcium-Sensing Receptor-Mediated Signaling. *J Bone Miner Res* **32**, 654-666, doi:10.1002/jbmr.3023 (2017).
78 Patat, O. *et al.* Truncating Mutations in the Adhesion G Protein-Coupled Receptor G2 Gene ADGRG2 Cause an X-Linked Congenital Bilateral Absence of Vas Deferens. *Am J Hum Genet* **99**, 437-442, doi:10.1016/j.ajhg.2016.06.012 (2016).

79 Sun, Y. *et al.* Optimization of a peptide ligand for the adhesion GPCR ADGRG2 provides a potent tool to explore receptor biology. *J Biol Chem*, doi:10.1074/jbc.RA120.014726 (2020).
80 Groot Kormelink, P. J. & Luyten, W. H. Cloning and sequence of full-length cDNAs encoding the human neuronal nicotinic acetylcholine receptor (nAChR) subunits beta3 and beta4 and expression of seven nAChR subunits in the human neuroblastoma cell line SH-SY5Y and/or IMR-32. *FEBS Lett* **400**, 309-314, doi:10.1016/s0014-5793(96)01383-x (1997).
81 Drayson, L. E. & Triplett, J. W. A Chrnb3-Cre BAC transgenic mouse line for manipulation of gene expression in retinal ganglion cells. *Genesis* **57**, e23305, doi:10.1002/dvg.23305 (2019).
82 Kiso, A. *et al.* Tolloid-Like 1 Negatively Regulates Hepatic Differentiation of Human Induced Pluripotent Stem Cells Through Transforming Growth Factor Beta Signaling. *Hepatol Commun* **4**, 255-267, doi:10.1002/hep4.1466 (2020).
83 Rhodes, K. E. & Fawcett, J. W. Chondroitin sulphate proteoglycans: preventing plasticity or protecting the CNS? *J Anat* **204**, 33-48, doi:10.1111/j.1469-7580.2004.00261.x (2004).
84 Nikolova, Y. S. *et al.* FRAS1-related extracellular matrix 3 (FREM3) single-nucleotide polymorphism effects on gene expression, amygdala reactivity and perceptual processing speed: An accelerated aging pathway of depression risk. *Front Psychol* **6**, 1377, doi:10.3389/fpsyg.2015.01377 (2015).
85 Wang, K. *et al.* Cell-Type-Specific Expression Pattern of Proton-Sensing Receptors and Channels in Pituitary Gland. *Biophys J* **119**, 2335-2348, doi:10.1016/j.bpj.2020.10.013 (2020).
86 Xu, J. *et al.* GPR68 Senses Flow and Is Essential for Vascular Physiology. *Cell* **173**, 762-775 e716, doi:10.1016/j.cell.2018.03.076 (2018).
87 Wiley, S. Z., Sriram, K., Salmeron, C. & Insel, P. A. GPR68: An Emerging Drug Target in Cancer. *Int J Mol Sci* **20**, doi:10.3390/ijms20030559 (2019).
88 Yang, S. *et al.* Cytochrome P-450 epoxygenases protect endothelial cells from apoptosis induced by tumor necrosis factor-alpha via MAPK and PI3K/Akt signaling pathways. *Am J Physiol Heart Circ Physiol* **293**, H142-151, doi:10.1152/ajpheart.00783.2006 (2007).
89 Liu, P. T. *et al.* Toll-like receptor triggering of a vitamin D-mediated human antimicrobial response. *Science* **311**, 1770-1773, doi:10.1126/science.1123933 (2006).
90 Werneburg, S. *et al.* Polysialylation at Early Stages of Oligodendrocyte Differentiation Promotes Myelin Repair. *J Neurosci* **37**, 8131-8141, doi:10.1523/JNEUROSCI.1147-17.2017 (2017).
91 Dennis, J., Waller, C., Timpl, R. & Schirrmacher, V. Surface sialic acid reduces attachment of metastatic tumour cells to collagen type IV and fibronectin. *Nature* **300**, 274-276, doi:10.1038/300274a0 (1982).
92 Mylonis, I., Simos, G. & Paraskeva, E. Hypoxia-Inducible Factors and the Regulation of Lipid Metabolism. *Cells* **8**, doi:10.3390/cells8030214 (2019).
93 Li, S. H. *et al.* An actin-binding protein ESPN is an independent prognosticator and regulates cell growth for esophageal squamous cell carcinoma. *Cancer Cell Int* **18**, 219, doi:10.1186/s12935-018-0713-x (2018).

94 Tan, Y. *et al.* The homeoprotein Dlx5 drives murine T-cell lymphomagenesis by directly transactivating Notch and upregulating Akt signaling. *Oncotarget* **8**, 14941-14956, doi:10.18632/oncotarget.14784 (2017).
95 Meyerhardt, J. A. *et al.* Netrin-1: interaction with deleted in colorectal cancer (DCC) and alterations in brain tumors and neuroblastomas. *Cell Growth Differ* **10**, 35-42 (1999).
96 Grandin, M. *et al.* Inhibition of DNA methylation promotes breast tumor sensitivity to netrin-1 interference. *EMBO Mol Med* **8**, 863-877, doi:10.15252/emmm.201505945 (2016).
97 Porter, L. F. *et al.* A role for repressive complexes and H3K9 di-methylation in PRDM5-associated brittle cornea syndrome. *Hum Mol Genet* **24**, 6565-6579, doi:10.1093/hmg/ddv345 (2015).
98 Yao, L. L. *et al.* Astrocytic neogenin/netrin-1 pathway promotes blood vessel homeostasis and function in mouse cortex. *J Clin Invest* **130**, 6490-6509, doi:10.1172/jci132372 (2020).
99 Wilson, L. H. *et al.* Liver Glycogen Phosphorylase Deficiency Leads to Profibrogenic Phenotype in a Murine Model of Glycogen Storage Disease Type VI. *Hepatol Commun* **3**, 1544-1555, doi:10.1002/hep4.1426 (2019).
100 Luo, X. *et al.* Novel PYGL mutations in Chinese children leading to glycogen storage disease type VI: two case reports. *BMC Med Genet* **21**, 74, doi:10.1186/s12881-020-01010-4 (2020).
101 Man, M. Q. *et al.* Basis for enhanced barrier function of pigmented skin. *J Invest Dermatol* **134**, 2399-2407, doi:10.1038/jid.2014.187 (2014).
102 Matesic, L. E. *et al.* Mutations in Mlph, encoding a member of the Rab effector family, cause the melanosome transport defects observed in leaden mice. *Proc Natl Acad Sci U S A* **98**, 10238-10243, doi:10.1073/pnas.181336698 (2001).
103 Robinson, C. L. *et al.* The adaptor protein melanophilin regulates dynamic myosin-Va:cargo interaction and dendrite development in melanocytes. *Mol Biol Cell* **30**, 742-752, doi:10.1091/mbc.E18-04-0237 (2019).
104 Mishra, N. K. *et al.* FXYD proteins stabilize Na,K-ATPase: amplification of specific phosphatidylserine-protein interactions. *J Biol Chem* **286**, 9699-9712, doi:10.1074/jbc.M110.184234 (2011).
105 Lubarski, I., Karlish, S. J. & Garty, H. Structural and functional interactions between FXYD5 and the Na+-K+-ATPase. *Am J Physiol Renal Physiol* **293**, F1818-1826, doi:10.1152/ajprenal.00367.2007 (2007).
106 Degrandi, D. *et al.* Extensive characterization of IFN-induced GTPases mGBP1 to mGBP10 involved in host defense. *J Immunol* **179**, 7729-7740, doi:10.4049/jimmunol.179.11.7729 (2007).
107 Degrandi, D. *et al.* Murine guanylate binding protein 2 (mGBP2) controls Toxoplasma gondii replication. *Proc Natl Acad Sci U S A* **110**, 294-299, doi:10.1073/pnas.1205635110 (2013).
108 Artegiani, B. *et al.* Tox: a multifunctional transcription factor and novel regulator of mammalian corticogenesis. *EMBO J* **34**, 896-910, doi:10.15252/embj.201490061 (2015).
109 Vorkapic, E., Kunath, A. & Wagsater, D. Effects of osteoprotegerin/TNFRSF11B in two models of abdominal aortic aneurysms. *Mol Med Rep* **18**, 41-48, doi:10.3892/mmr.2018.8936 (2018).

110 Cawley, K. M. *et al.* Local Production of Osteoprotegerin by Osteoblasts Suppresses Bone Resorption. *Cell Rep* **32**, 108052, doi:10.1016/j.celrep.2020.108052 (2020).

111 White, S. N. *et al.* Genome-wide association identifies multiple genomic regions associated with susceptibility to and control of ovine lentivirus. *PLoS One* **7**, e47829, doi:10.1371/journal.pone.0047829 (2012).

112 Kong, W., Longaker, M. T. & Lorenz, H. P. Molecular cloning and expression of keratinocyte proline-rich protein, a novel squamous epithelial marker isolated during skin development. *J Biol Chem* **278**, 22781-22786, doi:10.1074/jbc.M210488200 (2003).

113 Nissila, E. *et al.* C4B gene influences intestinal microbiota through complement activation in patients with paediatric-onset inflammatory bowel disease. *Clin Exp Immunol* **190**, 394-405, doi:10.1111/cei.13040 (2017).

114 Uyama, T., Kitagawa, H., Tamura Ji, J. & Sugahara, K. Molecular cloning and expression of human chondroitin N-acetylgalactosaminyltransferase: the key enzyme for chain initiation and elongation of chondroitin/dermatan sulfate on the protein linkage region tetrasaccharide shared by heparin/heparan sulfate. *J Biol Chem* **277**, 8841-8846, doi:10.1074/jbc.M111434200 (2002).

115 Mizumoto, S. *et al.* CSGALNACT1-congenital disorder of glycosylation: A mild skeletal dysplasia with advanced bone age. *Hum Mutat* **41**, 655-667, doi:10.1002/humu.23952 (2020).

116 Onishi, K. & Zou, Y. Sonic Hedgehog switches on Wnt/planar cell polarity signaling in commissural axon growth cones by reducing levels of Shisa2. *Elife* **6**, doi:10.7554/eLife.25269 (2017).

117 Desai, B. S., Chadha, A. & Cook, B. The stum gene is essential for mechanical sensing in proprioceptive neurons. *Science* **343**, 1256-1259, doi:10.1126/science.1247761 (2014).

118 Chen, C., Jiang, L., Zhang, Y. & Zheng, W. FOXA1-induced LINC01207 facilitates head and neck squamous cell carcinoma via up-regulation of TNRC6B. *Biomed Pharmacother* **128**, 110220, doi:10.1016/j.biopha.2020.110220 (2020).

119 Plun-Favreau, H. *et al.* The ciliary neurotrophic factor receptor alpha component induces the secretion of and is required for functional responses to cardiotrophin-like cytokine. *EMBO J* **20**, 1692-1703, doi:10.1093/emboj/20.7.1692 (2001).

120 Cox-Limpens, K. E., Vles, J. S., D, L. A. v. d. H., Zimmermann, L. J. & Gavilanes, A. W. Fetal asphyctic preconditioning alters the transcriptional response to perinatal asphyxia. *BMC Neurosci* **15**, 67, doi:10.1186/1471-2202-15-67 (2014).

121 Katsu, Y. & Iguchi, T. Tissue-specific expression of Clec2g in mice. *Eur J Cell Biol* **85**, 345-354, doi:10.1016/j.ejcb.2005.12.004 (2006).

122 Ekici, A. B. *et al.* Disturbed Wnt Signalling due to a Mutation in CCDC88C Causes an Autosomal Recessive Non-Syndromic Hydrocephalus with Medial Diverticulum. *Mol Syndromol* **1**, 99-112, doi:10.1159/000319859 (2010).

123 Ear, J. *et al.* Tyrosine-Based Signals Regulate the Assembly of Daple·PARD3 Complex at Cell-Cell Junctions. *iScience* **23**, 100859, doi:10.1016/j.isci.2020.100859 (2020).

124 Shastry, S. *et al.* Deletion in the A4GALT Gene Associated with Rare "P null" Phenotype: The First Report from India. *Transfus Med Hemother* **47**, 186-189, doi:10.1159/000501916 (2020).

125 Gao, S. *et al.* The interaction between flagellin and the glycosphingolipid Gb3 on host cells contributes to Bacillus cereus acute infection. *Virulence* **11**, 769-780, doi:10.1080/21505594.2020.1773077 (2020).
126 Giessner, C. *et al.* Vnn1 pantetheinase limits the Warburg effect and sarcoma growth by rescuing mitochondrial activity. *Life Sci Alliance* **1**, e201800073, doi:10.26508/lsa.201800073 (2018).
127 Ferreira, D. W., Naquet, P. & Manautou, J. E. Influence of Vanin-1 and Catalytic Products in Liver During Normal and Oxidative Stress Conditions. *Curr Med Chem* **22**, 2407-2416, doi:10.2174/0929867322220150722124307 (2015).
128 Punetha, J. *et al.* Biallelic CACNA2D2 variants in epileptic encephalopathy and cerebellar atrophy. *Ann Clin Transl Neurol* **6**, 1395-1406, doi:10.1002/acn3.50824 (2019).
129 Lodge, E. J. *et al.* Requirement of FAT and DCHS protocadherins during hypothalamic-pituitary development. *JCI Insight* **5**, doi:10.1172/jci.insight.134310 (2020).
130 Karrys, A. *et al.* Bioactive Dietary VDR Ligands Regulate Genes Encoding Biomarkers of Skin Repair That Are Associated with Risk for Psoriasis. *Nutrients* **10**, doi:10.3390/nu10020174 (2018).
131 Pai, Y. J. *et al.* Glycine decarboxylase deficiency causes neural tube defects and features of non-ketotic hyperglycinemia in mice. *Nat Commun* **6**, 6388, doi:10.1038/ncomms7388 (2015).
132 Diaz-Horta, O. *et al.* Ripor2 is involved in auditory hair cell stereociliary bundle structure and orientation. *J Mol Med (Berl)* **96**, 1227-1238, doi:10.1007/s00109-018-1694-x (2018).
133 Huang, X. *et al.* NOV/CCN3 induces cartilage protection by inhibiting PI3K/AKT/mTOR pathway. *J Cell Mol Med* **23**, 7525-7534, doi:10.1111/jcmm.14621 (2019).
134 Kuwahara, M. *et al.* CCN3 (NOV) Drives Degradative Changes in Aging Articular Cartilage. *Int J Mol Sci* **21**, doi:10.3390/ijms21207556 (2020).
135 Sreedharan, S. *et al.* The G protein coupled receptor Gpr153 shares common evolutionary origin with Gpr162 and is highly expressed in central regions including the thalamus, cerebellum and the arcuate nucleus. *FEBS J* **278**, 4881-4894, doi:10.1111/j.1742-4658.2011.08388.x (2011).
136 Li, P. *et al.* Novel Therapy for Glioblastoma Multiforme by Restoring LRRC4 in Tumor Cells: LRRC4 Inhibits Tumor-Infitrating Regulatory T Cells by Cytokine and Programmed Cell Death 1-Containing Exosomes. *Front Immunol* **8**, 1748, doi:10.3389/fimmu.2017.01748 (2017).
137 Zhao, C. *et al.* LRRC4 Suppresses E-Cadherin-Dependent Collective Cell Invasion and Metastasis in Epithelial Ovarian Cancer. *Front Oncol* **10**, 144, doi:10.3389/fonc.2020.00144 (2020).
138 Seda, O. *et al.* ZBTB16 and metabolic syndrome: a network perspective. *Physiol Res* **66**, S357-S365, doi:10.33549/physiolres.933730 (2017).
139 He, H. *et al.* Study on the mechanism behind lncRNA MEG3 affecting clear cell renal cell carcinoma by regulating miR-7/RASL11B signaling. *J Cell Physiol* **233**, 9503-9515, doi:10.1002/jcp.26849 (2018).
140 Ligthart-Melis, G. C. & Deutz, N. E. Is glutamine still an important precursor of citrulline? *Am J Physiol Endocrinol Metab* **301**, E264-266, doi:10.1152/ajpendo.00223.2011 (2011).

141 Hannemann, J., Zummack, J., Hillig, J. & Boger, R. Metabolism of asymmetric dimethylarginine in hypoxia: from bench to bedside. *Pulm Circ* **10**, 2045894020918846, doi:10.1177/2045894020918846 (2020).
142 Hannemann, J. *et al.* Upregulation of DDAH2 Limits Pulmonary Hypertension and Right Ventricular Hypertrophy During Chronic Hypoxia in Ddah1 Knockout Mice. *Front Physiol* **11**, 597559, doi:10.3389/fphys.2020.597559 (2020).

Supplementary Figure Legends

Supplementary Figure 1. Distribution of normalized and variance stabilized expression values across individual sequencing libraries for three mice (M1-M3) of each respective genotype. A, Gene expression estimated as expected number of Fragments Per Kilobase of transcript x 10^6 Mapped reads (FPKM, box plot). The box plot for each region indicates five statistics (top to bottom: maximum, upper quartile, median, lower quartile and minimum). B, Principal component analysis (PCA). Corneal RNA gene expression from three mice (M1-M3) of each respective genotype, *slc4a11*$^{-/-}$ and *slc4a11*$^{-/}$, was subjected to PCA. Results of exploratory analysis were visualized as principal component plots. PCA was performed on the 1500 of genes with the highest median absolute deviation.

Supplementary Figure 2. Differentially expressed genes in mouse cornea. Cluster analysis (hierarchical clustering) of expression patterns of differentially expressed genes between corneas of *slc4a11*$^{+/+}$ and *slc4a11*$^{-/-}$ mice. Heatmap represents normalized and variance stabilized values of differentially expressed genes (adjusted p value < 0.05 and log2 fold change > 0.59). Both columns (samples) and rows (genes) underwent hierarchical non-supervised clustering using Euclidean distance and ward.D clustering method. Magnitude of expression differences (Row Z-score) are indicated by different colors in the heat map from red (large increase in relative expression) to green (large decrease in relative expression).

Supplementary Figure 3. Gene clustering for *slc4a11*$^{+/+}$ and *slc4a11*$^{-/-}$ mice. Cluster analysis of expression patterns of differentially expressed genes for three mice (M1-M3) of each indicated

genotype. The non-supervised hierarchical clustering of the top 1500 genes detected with the highest median absolute deviation is shown by heat map. Expression changes were represented by the indicated color scale. Dendrograms corresponding to different groups are presented by brackets (*top*).

Supplementary Figure 4 Principal component analysis clustered the patterns of gene expression by tissue type. Gene expression data from RNAseq were processed to normalize transcripts in mouse cornea. Expression of these genes was compared to expression levels of these genes in a panel of 13 indicated mouse tissues (from tissue expression database, https://www.omicsdi.org/dataset/arrayexpress-repository/E-MTAB-6081).

Supplementary Figure 5. Specificity of qReal-time RT-PCR. **A**, Agarose gel (3%) showing amplification of a specific PCR product for each gene tested in this study, for *slc4a11*$^{+/+}$ and *slc4a11*$^{-/-}$ mice corneal cDNA. Samples were run on separate agarose gels, as indicated by white breaks between sections. **B**, Agarose gel (3%) of amplified col14a1 for *slc4a11*$^{+/+}$ and *slc4a11*$^{-/-}$ mice corneal cDNA; **C**, Melting curves analysis of *tubb4a* and *espn* products. The negative first derivative of the change in fluorescence is plotted as a function temperature in °C (-df/dt). Full, uncropped versions of all gels are in Suppl. Fig. 6.

Supplementary Figure 6. Full length, unedited versions of images from Fig. 5. **A**, Agarose gel (3%) showing amplification of a specific PCR product for each gene tested in this study, for *slc4a11*$^{+/+}$

and *slc4a11*$^{-/-}$ mice corneal cDNA. Samples were run on separate agarose gels. **B**, Agarose gel (3%) of amplified *col14a1* for *slc4a11*$^{+/+}$ and *slc4a11*$^{-/-}$ mice corneal cDNA. NRS, non relevant sample (these samples were not analyzed in the current manuscript).

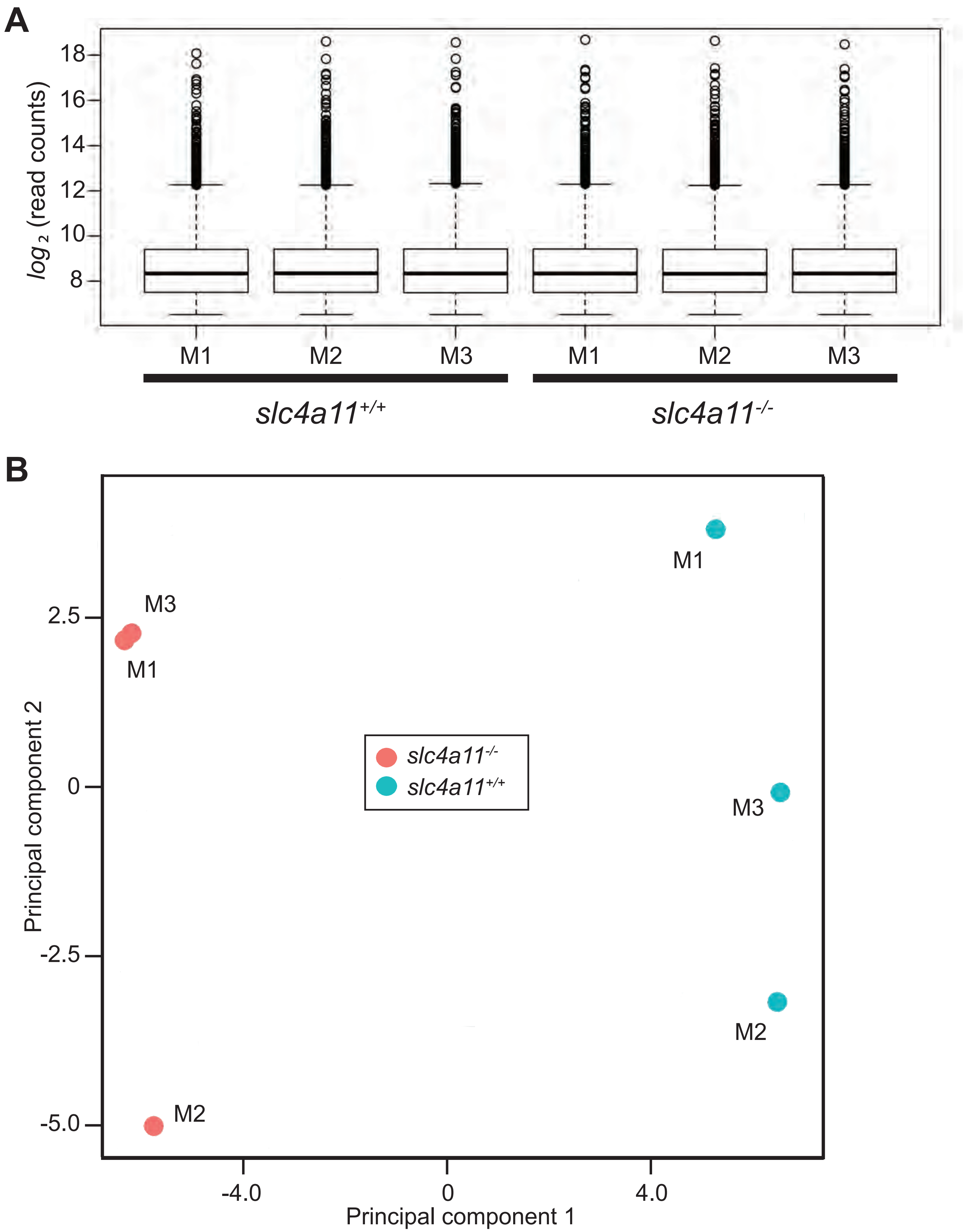

Suppl. Figure 1

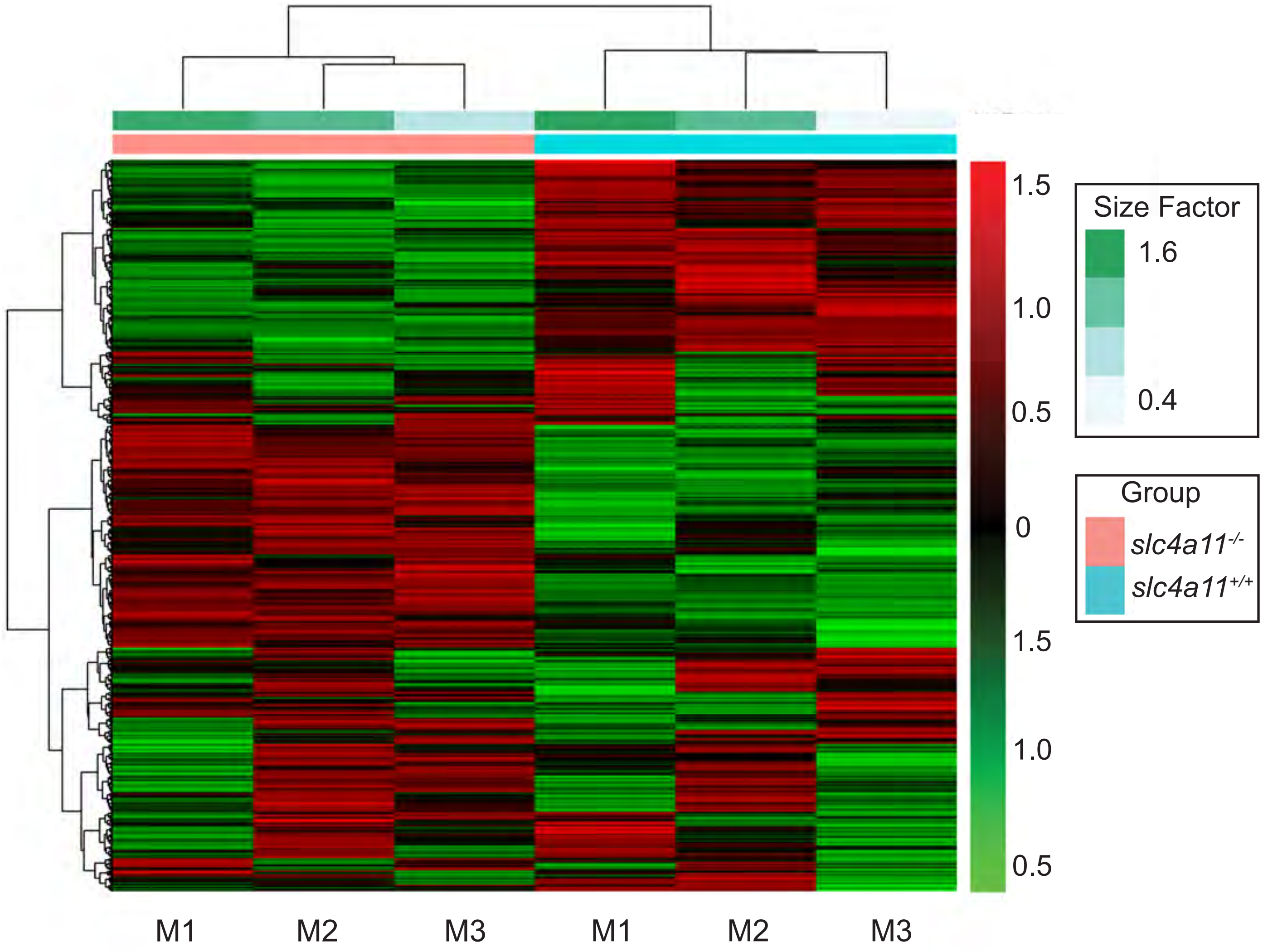

Suppl. Figure 2

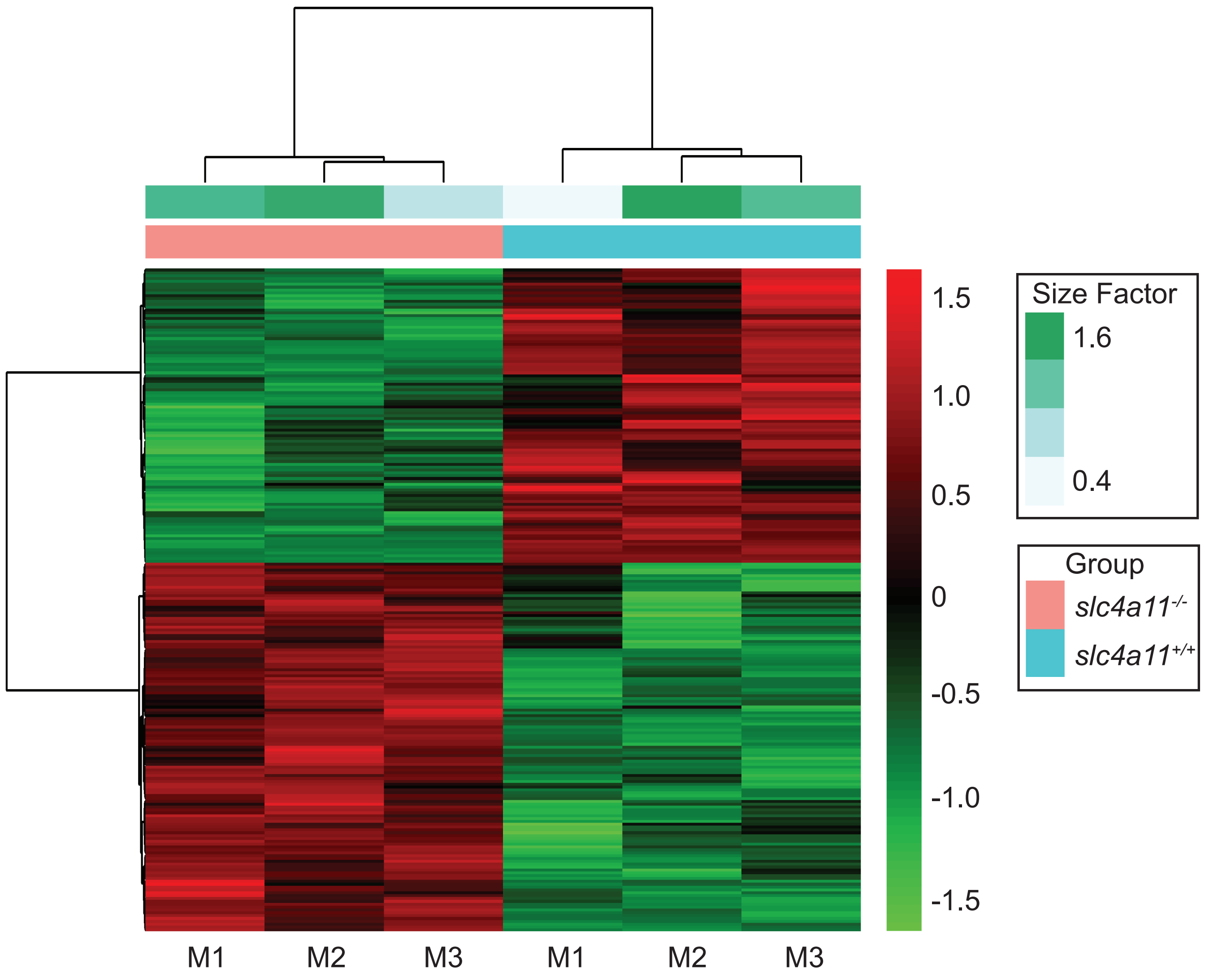

Suppl. Figure 3

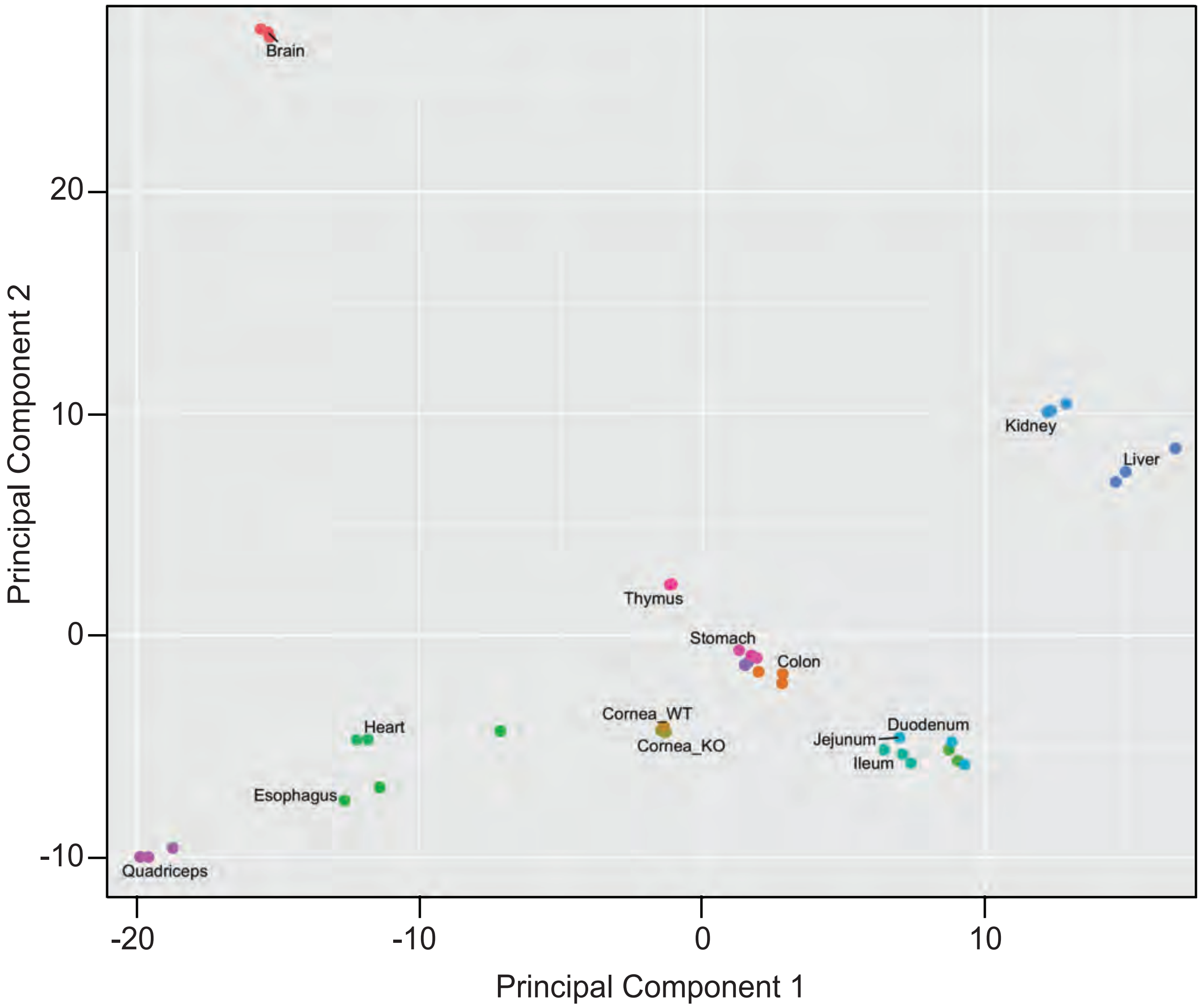

Suppl. Figure 4

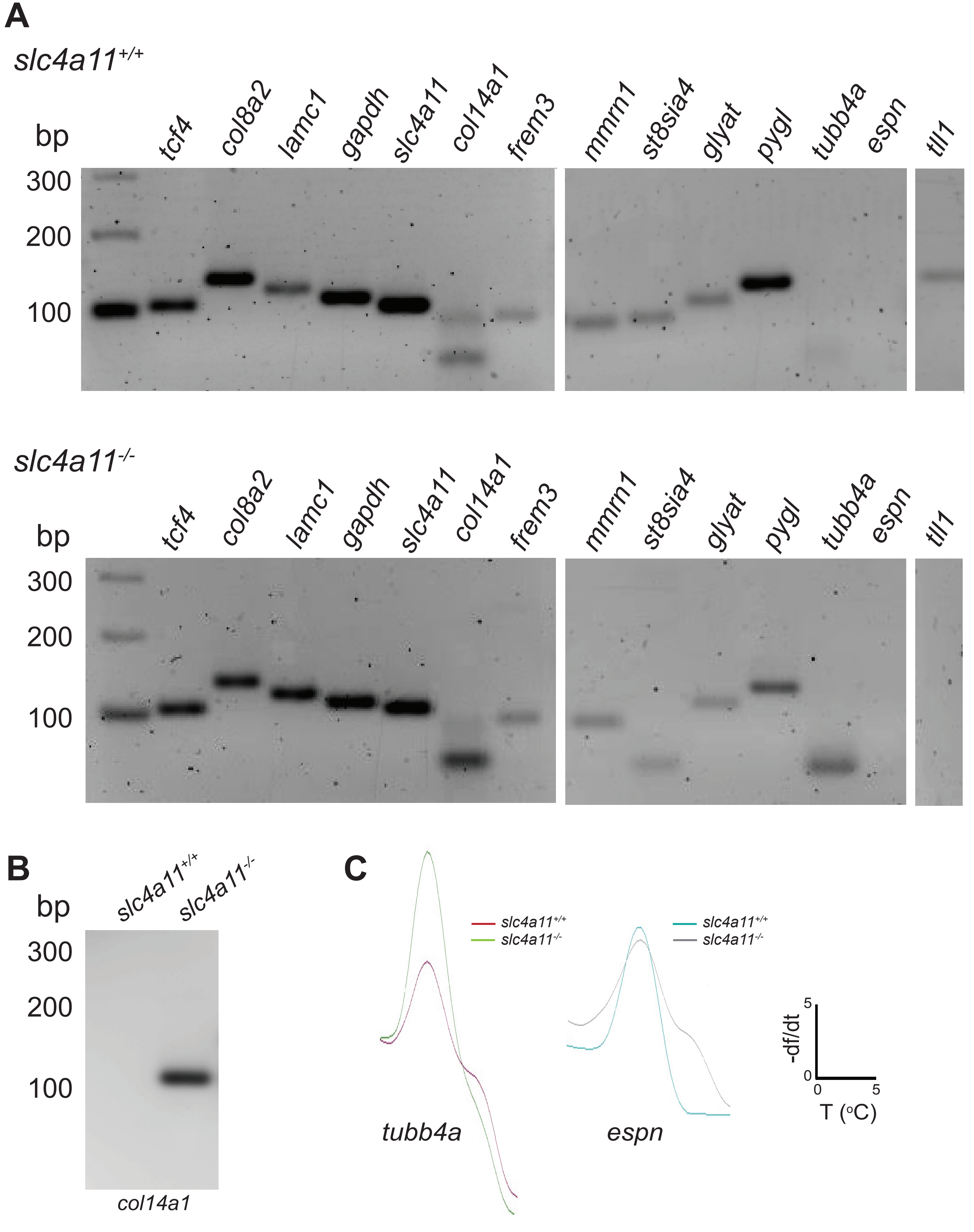

Suppl. Figure 5

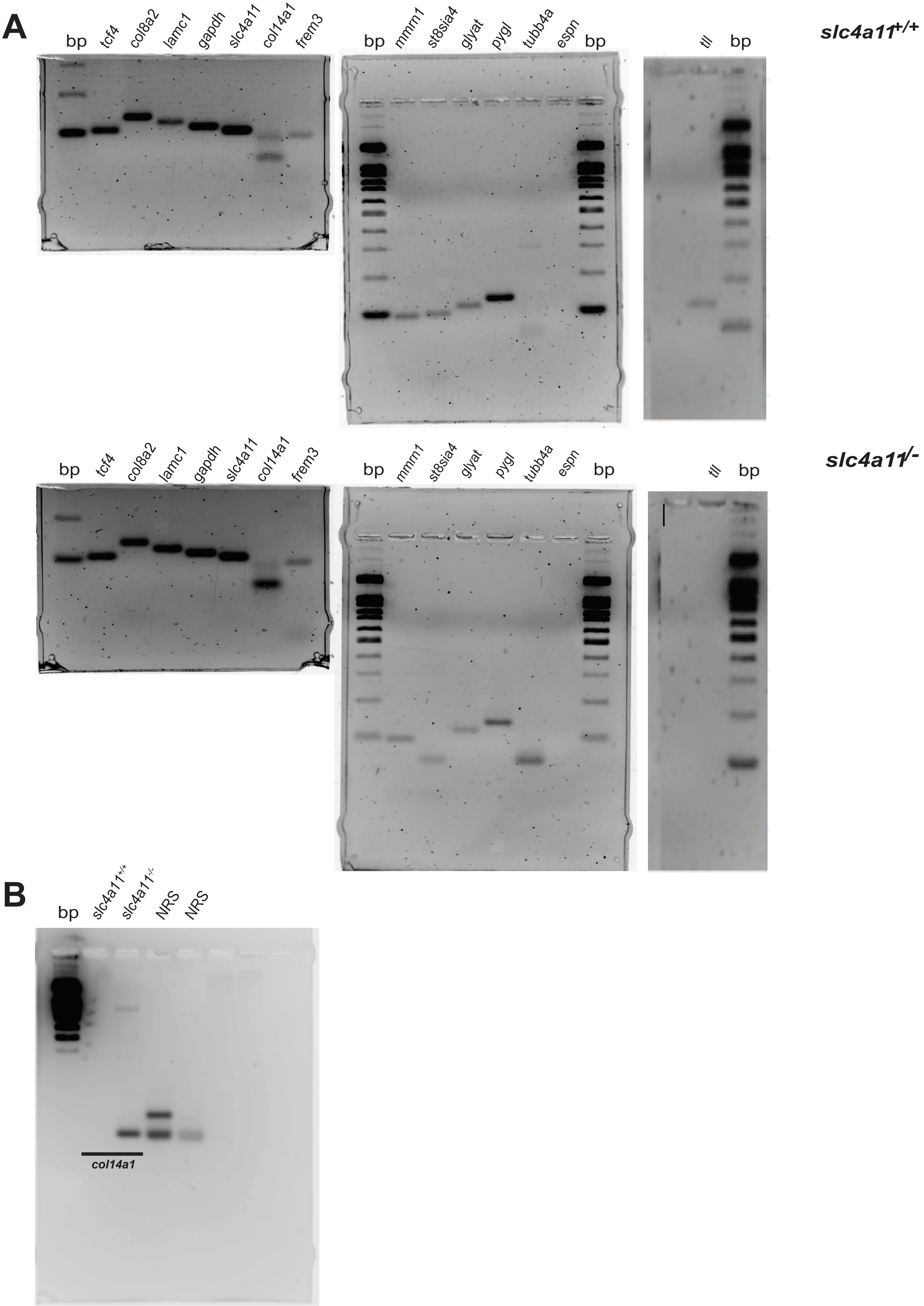

Suppl. Figure 6
